# Structural Features of Nerve Guidance Conduits and Scaffolds in Preventing Axonal Misdirection: A Systematic Review of Retrograde Tracing Studies

**DOI:** 10.3390/bioengineering13020220

**Published:** 2026-02-13

**Authors:** Aleksa Mićić, Milan Aksić, Andrija Savić, Joko Poleksić, Jovan Grujić, Milan Lepić, Dubravka Aleksić, Lazar Vujić, Lukas Rasulić

**Affiliations:** 1 Faculty of Medicine, University of Belgrade, 11000 Belgrade, Serbia; aleksamicic.md@gmail.com (A.M.);; 2 Clinic for Neurosurgery, University Clinical Centre of Serbia, 11000 Belgrade, Serbia; 3 Institute of Human Anatomy ‘’Niko Miljanic’’, Faculty of Medicine, University of Belgrade, 11000 Belgrade, Serbia; 4 Faculty of Medicine, University of Defence, 11000 Belgrade, Serbia; 5 Clinic for Neurosurgery, Military Medical Academy, 11000 Belgrade, Serbia

**Keywords:** nerve regeneration, axonal misdirection, artificial nerve grafts, nerve guidance conduits, nerve scaffolds, retrograde tracing

## Abstract

**Background:** Axonal misdirection remains a major limitation in peripheral nerve repair. While nerve guidance conduits (NGCs) and nerve scaffolds (NSCs) have advanced structurally, it is unclear whether these designs effectively reduce misdirection compared to autografts (ANGs). This systematic review evaluates the impact of NGC and NSC structural features on axonal dispersion and reinnervation accuracy using retrograde tracing animal models. **Methods:** A systematic search was performed through Medline (PubMed), Scopus (EBSCOhost), and the Cochrane Library from inception to December 2024. Eligible studies included mammalian in vivo models of peripheral nerve transection repaired by direct coaptation, autografts, or artificial conduits and assessed with retrograde axonal tracing. Data on neurons labeling, innervation accuracy, and histomorphometric parameters were extracted, and misdirection rates were calculated. Risk of bias was assessed using the SYRCLE tool. Due to heterogeneity, data were synthesized narratively following the SWiM framework. **Results:** Out of 4043 records identified through database searching and 37 through citation searching, 19 studies (49 experimental groups) met the inclusion criteria. Motoneuron counts were consistently reported across all arms, but no outcome assessing axonal misdirection was reported in more than half. Structured designs resulted in outcomes more closely aligned with ANG repair, while unstructured generally underperformed, and certainty of evidence was very low. **Discussion:** The evidence in this study was limited by high risk of bias, substantial inconsistency across heterogeneous study designs and outcomes, and imprecision from small animal models with sparse outcome measures. Despite the trend for structured designs to improve over basic hollow designs, current evidence does not support any structure as superior. Future research should be more standardized to provide reliable knowledge translational into clinical practice.

## 1. Introduction

Peripheral nerve injuries may lead to serious functional deficits that significantly impact the quality of life of the affected individuals [1,2]. In cases of insufficient spontaneous recovery, a range of multi-disciplinary interventions may be implemented across the three divided action areas, including stimulating neural growth, treating the site of nerve injury, and maintaining the viability of the target organ [3,4]. Any failure to fulfill these criteria can have a significant impact on the final functional recovery.

In cases with a loss of nerve continuity, surgical repair of the injury site presents the primary treatment option [5]. Direct end-to-end (ETE) nerve coaptation without tension has historically been regarded as the gold standard due to its high success rates. However, in cases when tension-free coaptation is not feasible, biologic nerve grafts (BNGs), such as autologous nerve grafts (ANGs) remain the primary surgical alternative. Successfully repairing nerve defects by grafting and ensuring complete functional recovery remains a great challenge, particularly in extensive or proximal nerve injuries considered irreparable [6,7]. Although ANGs still stand as the gold standard for nerve defect repair [8], their limitations cannot be overlooked [9]. Therefore, substantial resources are devoted to advancing the development of artificial nerve grafts (ArtNGs), primarily in the form of tubular nerve guidance conduits (NGCs) or nontubular three-dimensional nerve scaffolds (NSCs) [10,11].

The main strategies in ArtNG development include the selection of the material, the fabrication method, the structural design, and functional enhancement [12,13]. Due to the failure of early ArtNG designs to ensure aligned axonal guidance over nerve gap sizes comparable to ANGs, most studies shifted their focus toward bridging over gap sizes similar to ANGs. Given the cellular and biochemical structure of ANGs which has been shown to promote axonal guidance toward distal nerve stumps most studies have concentrated on enhancing ArtNGs by integrating a variety of molecular, cellular, and physical factors and advancing biomaterial characteristics [14,15,16,17,18,19].

Although many studies have demonstrated that ArtNGs can support nerve fiber growth and facilitate regeneration towards the distal stump, simply reaching the distal end does not guarantee precise target reinnervation or the restoration of voluntary function. Most of the literature selectively reports morphological and electrophysiological outcomes, frequently presenting ArtNG repair as numerically comparable to ANG repair and emphasizing its beneficial impact on peripheral nerve regeneration. Meanwhile, it rarely assesses axonal misdirection in terms of axonal dispersion and aberrant reinnervation. Such interpretations lack deeper insight, and outcomes remain largely incomparable due to unstandardized reporting methods. Even when direct comparisons are possible, the correlations between reported outcomes generally remain weak [20,21,22,23,24,25].

**Figure 1:**
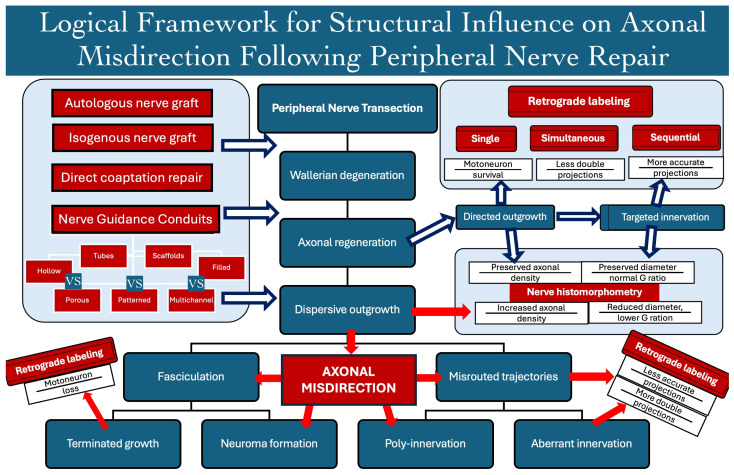
Logical model of the biological and structural determinants of axonal misdirection following peripheral nerve transection and repair. Evaluation methods such as retrograde axonal labeling (single, simultaneous, or sequential) assess motoneuron (MN) survival, double projections and reinnervation precision, and when combined with nerve morphometry (axonal density, diameter, and G-ratio), the model illustrates how the repair strategy, structural design, and evaluation modality interact to shape the regeneration quality and functional outcomes [49,50,51,52,53].

Axonal misdirection (misrouting) represents its failure to reach the corresponding target organs, leading to their terminated growth or mismatched target organ reinnervation (Figure 1). It occurs due to the dispersive axonal outgrowth, its tendency for selective reinnervation, and its variable intraneural branching topography. Terminated axonal growth may involve adhesion to surrounding tissues, fasciculation, and neuroma formation. Mismatched reinnervation may involve motor axons spreading into antagonistic muscle groups, sensitive pathways, or both, as well as sensory axons spreading into muscle pathways or inappropriate sensory organs. For a long time, axonal misdirection was recognized as a significant factor contributing to poor outcomes following nerve repair, particularly in extensive injuries with large structural defects. With the advance of axonal tracing techniques, there is emerging evidence linking axonal misdirection with poor outcomes following nerve repair [26,27,28,29,30,31,32,33,34,35,36,37,38,39,40,41,42,43,44,45,46,47,48].

Despite the significance of manipulating molecular, cellular and genetic factors to provide proper axonal guidance and innervation accuracy, only a suitable ArtNG structural configuration may have a significant impact on axonal misdirection, especially in larger nerve gaps [54,55]. The research aimed at improving conduit performance by emphasizing functional properties usually involves few enhanced structural designs and compares their impact with ANGs, while lacking an assessment of the isolated impact of the structural design and comparing how different structures with the same functional enhancement impact axonal guidance. Consequently, the scientific community lacks sufficient comparative data on structural features and their role in preventing axonal regeneration and guidance [56,57,58,59].

Most of the recently published review articles focus on functionally enhanced ArtNGs while reviews conducted on designs and fabrication strategies usually briefly cover the topic, referring to other review articles or few research studies [13,60,61]. To the best of our knowledge, no systematic review has comprehensively examined the role of ArtNG structural design in influencing axonal misdirection. Without systematically assessing and comparing axonal misdirection across various ArtNG structures, it remains challenging to identify optimal designs capable of truly matching or surpassing the functional efficacy of ANGs.

This paper aims to fill this gap by systematically reviewing various NGC and NSC design strategies with a specific focus on their potential to influence axonal misdirection. By evaluating the effectiveness of different structural features, this review will highlight key challenges and future perspectives in ArtNG optimization for the improvement of nerve regeneration and recovery.

## 2. Materials and Methods

A systematic review was conducted and last searched on 1 January 2025 in accordance with the PRISMA 2020 statement. The full PRISMA checklist is provided as a Supplementary Materials [62]. The review protocol was written a priori but was not registered; interested readers may obtain a copy from the corresponding author.

### 2.1. Eligibility Criteria

The eligibility criteria were defined using the PICO framework and are summarized in Table 1. The review includes original, peer-reviewed studies involving mammalian in vivo models with full-text availability, published in English up to the end of 2024. Only studies involving experimental groups of peripheral nerve transection injury and immediate repair using functionally non-enhanced ArtNGs in comparison with direct ETE coaptation, BNGs, or structurally different ArtNGs were considered.

To ensure the assessment of axonal misdirection, only studies that implemented retrograde axonal tracing were included. This technique was considered essential, as it is emphasized by numerous authors as the most reliable method for assessing both axonal dispersion and reinnervation accuracy following peripheral nerve repair [30,47,63,64]. A more detailed explanation of the selected outcomes is provided in the Data Items (Outcome) section (Section 2.5).

To ensure homogeneity, internal validity, and relevance to the objective of assessing the structural features of NGCs and NSCs on axonal misdirection, specific exclusion criteria were applied at the population, intervention, comparator, and outcome levels.

#### 2.1.1. Population Exclusions

Studies involving juvenile, elderly, or genetically altered animals were excluded due to their altered regenerative capacity, which could confound comparisons between conduit designs. Models with bilateral or multiple nerve injuries or involving proximal neural structures (central motoneurons, cranial nerves, spinal cord, spinal root or nerve), were excluded because they introduce systemic or anatomical complexities unrelated to peripheral nerve conduit performance. Non-transection injury models (crush, compression, freeze, radiation, or metabolic) were excluded because they differ fundamentally in pathophysiology and regeneration dynamics from clean transection injuries. Studies that included fewer than three animals per experimental group or had a short-term follow-up period were excluded, as such designs are unlikely to provide sufficient data for valid synthesis or reliable evaluation of reinnervation outcomes.

#### 2.1.2. Intervention Exclusions

This review focused on early repair using linear, structurally distinct ArtNGs. Studies using delayed repair, indirect techniques (nerve transfers, end-to-side (ETS) or interfascicular coaptation), or intentional misalignment at the suture site were excluded due to their divergent regenerative mechanisms. Combined interventions and two-stage procedures were excluded to avoid confounding effects from multimodal treatments. To isolate structural effects, individualized or custom-made conduits, non-linear designs (di-branched, multi-branched), and functionally enhanced ArtNGs (filled with cells, adhesive molecules, responsive hydrogels, growth factors, or made of conductive materials) were also excluded. Biological grafts such as cellular soft tissues (muscle, vein, omentum) or acellular grafts (auto-, allo-, or xenografts) were excluded, as they confound the assessment of structural design alone.

#### 2.1.3. Comparator Exclusions

Eligible studies were required to include at least two distinct interventions (ETE/BNG vs. ArtNG) to allow a direct comparison. Studies comparing ArtNGs with identical structures made of different materials without isolating structural variables were excluded, as they did not address the primary aim of this review.

#### 2.1.4. Outcome Exclusions

Studies were excluded if they did not provide sufficient outcome details to assess axonal misdirection. This included qualitative-only presentations such as textual interpretations of statistical significance without raw data, microscopy images with size bars but without quantitative analysis, and graphical charts lacking standard deviations or error bars. Studies using only single axonal tracing without nerve histomorphometry, or vice versa, were excluded. Short-term follow up was excluded due to insufficient time for reinnervation of the muscles and assessment using axonal tracing.

### 2.2. Information Sources and Search Strategy

PubMed (MEDLINE^®^), Scopus (via EBSCOhost), and the Cochrane Library were searched from inception to 31 December 2024.

A comprehensive list of keywords and MeSH terms was developed to reflect the terminology used in peripheral nerve repair and tissue engineering (Table A1). Filters applied across all platforms included the peer-reviewed publication type, full-text availability, the exclusion of reviews, systematic reviews, and meta-analyses, as well as articles mentioning “spinal cord”, “brain,” or “stroke” in the title field (Table A2).

The PubMed search strategy was constructed step-by-step (Table S1) and adapted for Scopus via the EBSCOhost interface using prompt-based assistance from ChatGPT (GPT-4, OpenAI, San Francisco, CA, USA) (Table S2). The SmartText search option was used in EBSCOhost, with peer-reviewed and full-text filters enabled. The Cochrane Library was also searched using the same strategy; no eligible studies were retrieved.

Additionally, a supplementary search in PubMed (MEDLINE^®^) was performed using the same search query but filtered for review articles, systematic reviews, and meta-analyses, to support contextualization in the Introduction and Discussion. During full-text screening, the reference lists of all included studies were manually reviewed to identify additional eligible reports.

### 2.3. Selection Process

All search results were imported into EndNote X20, where duplicate records were automatically identified and removed. Title and abstract screening was performed independently by two reviewers, using the predefined eligibility criteria. Semiautomated title screening was conducted using EndNote X20. Articles were grouped and excluded based on title keywords deemed irrelevant to the review objectives. This process was carried out by applying custom grouping and search rules within EndNote’s reference management system. Although automation was used to reduce the initial screening burden, manual verification was performed by same reviewers to ensure that potentially eligible studies were not inadvertently excluded. No external machine learning classifier was applied. Full-text articles were retrieved for all potentially eligible studies and evaluated in duplicate by the same reviewers. Discrepancies between reviewers at any stage were resolved through discussion. When disagreements persisted, a third independent reviewer adjudicated the decision.

If additional clarification of study details was needed, no attempts were made to contact the corresponding authors, and the studies were excluded but cited in the Excluded Studies section (Section 3.2). No automation tools, machine learning classifiers, or crowdsourcing methods were used in the study selection process. All records were screened manually. No translations were required during screening, as all included studies were published in English.

### 2.4. Data Collection

Data extraction was performed independently by two reviewers using a predefined piloted extraction form, with discrepancies resolved through discussion and confirmed by a third reviewer. When outcomes were presented only in graphical form, numerical data were extracted using WebPlotDigitizer v5, with interpretive and calculating assistance from ChatGPT (GPT-4, OpenAI, San Francisco, CA, USA). No other automation or translation tools were used in the data collection process.

### 2.5. Data Items (Outcomes)

In line with the primary objective of this study, which was to assess the structural impact of NGCs and NSCs on preventing axonal misdirection, the outcomes were selected to capture both axonal dispersion and reinnervation accuracy, recognized as the two principal indicators of misdirected regeneration. The frequently observed lack of correlation between commonly reported outcomes in experimental peripheral nerve repair research is largely attributed to the misdirected reinnervation of target organs. Therefore, retrograde axonal tracing has been emphasized as the most reliable method for assessing this phenomenon. Even though not without limitations [50,53], and with an awareness of the functional methods evolving to assess reinnervation accuracy, as well as the possibility of using immunostaining to evaluate axonal dispersion, retrograde axonal tracing currently appears to be the only viable technique capable of simultaneously assessing both axonal dispersion and reinnervation accuracy [63].

Therefore, the primary outcome sought for this study was the total number of retrogradely labeled motoneurons (LMNs). However, the sole use of LMN counts was considered insufficient for assessing axonal misdirection. Lower LMN values may result from motoneuron death rather than dispersion, while equal or even higher LMN counts compared to uninjured controls may reflect the significant misdirection of motor axons toward inappropriate targets. Such misrouting can yield misleadingly high LMN totals without translating into accurate reinnervation or functional recovery. Accordingly, the total LMN counts were always interpreted in relation to other metrics, which were selected secondary outcomes:

- LMN/LSN ratio—LMN counts relative to the number of retrogradely labeled sensory neurons (LSNs). The LMN/LSN ratio is defined as the proportion of labeled motoneurons to labeled sensory neurons, with lower ratios indicating higher misdirection of motor axons.
- TLN/TF ratio—total number of labeled motoneurons (TLN) relative to the total number of myelinated fibers (TF). To ensure comparability across studies, TF was standardized as a regenerated nerve (RN) measure, defined as values obtained at the mid-graft or distal graft; when both were reported, the mean value was used.
- Incorrect projections—The number of double or multiple labeled motoneurons following simultaneous retrograde tracing studies, expressed as a percentage of incorrectly projected neurons, indicating axonal dispersion, in the form of distal arborization and consequential polyinnervation and/or missinervation.
- Accurate projections—The number of double labeled motorneurons following sequential retrograde tracing studies, expressed as a percentage of correctly projecting neurons.
- AD/FD ratio—the relationship between axon diameter (AD) and fiber density (FD), where lower values reflect dense networks of fine sprouts indicative of axonal dispersion, and higher values reflect sparser networks of more mature fibers. Both AD and FD were standardized as RN measures, taken at the mid- or distal graft, or averaged when both sites were reported.
- TF ratios between nerve segments (e.g., MG.TF/PDS.TF, DDS.TF/MG.TF, DDS.TF/PDS.TF) indicating the extent of axonal continuity, dispersion, or loss.

All reported results that were compatible with outcome domains were sought. When studies presented multiple time points, the one closest to mid-term follow-up was prioritized, as this interval represents a standard endpoint in retrograde axonal tracing studies where axonal regeneration and target reinnervation are typically complete. Mid-term follow-up was defined as about 12 weeks in rodents, 20 weeks in rabbits, 28 weeks in cats and dogs, 32 weeks in sheep, goats, and pigs, and 38 weeks in non-human primates.

If outcome data were not uniformly reported, selection was guided by the methodological completeness and alignment with the defined concepts of axonal dispersion and reinnervation accuracy. Where numeric data were missing but derivable, values were calculated using ChatGPT (o4-Mini-High, OpenAI) and established formulas with standard deviation (SD) incorporated into every estimate and reported as approximate. SD was estimated from error bars by dividing the error bar value by the square root of the sample size (n). TF number was obtained by multiplying fiber density by the nerve cross-sectional area, with area expressed in mm^2^ or µm^2^ to match density units. FD was calculated as the fiber count divided by the analyzed area and is typically reported as fiber per mm^2^. AD was derived from available parameters using one of three approaches: subtracting twice the myelin thickness from the myelinated fiber diameter; multiplying the fiber diameter by the G-ratio (where G-ratio = axon diameter / fiber diameter); or, in cases where only G-ratio and myelin thickness were reported, calculating AD as the G-ratio multiplied by two times the myelin thickness divided by one minus the G-ratio. This approach ensured consistent interpretation across studies and allowed the integration of structurally relevant indices even when not explicitly reported.

### 2.6. Data Items (Other Variables)

In addition to outcomes, the following variables were extracted to describe the characteristics of the included studies and to support subgroup and sensitivity analyses:

- Study identifiers: Including the first author, year of publication, journal name, and funding.
- Population characteristics: Genus (strain), sex, weight, number of animals per group, injured nerve, length of nerve gap (mm).
- Intervention characteristics: Autologous/isogenous nerve graft orientation, artifitial nerve graft material and fabrication method, porosity, degradability, permeability, internal structure.
- Retrograde tracing methodology: The tracer compounds used, labeled nerves, and whether single, double, triple simultaneous or sequential labeling is performed.

### 2.7. Study Risk of Bias Assessment

The risk of bias was assessed using the SYRCLE Risk of Bias (RoB) tool, developed specifically for animal intervention studies [65]. The tool evaluates ten domains: sequence generation, baseline characteristics, allocation concealment, random housing, caregiver blinding, outcome assessment randomization, assessor blinding, incomplete outcome data, selective reporting, and other sources of bias. Two reviewers independently evaluated each included study, and discrepancies were resolved by discussion. A third reviewer verified final judgments. To support standardized and unbiased data handling, the tabulation and visual presentation of RoB results were performed using OpenAI’s GPT-5, a large language model trained on biomedical data and capable of automated evidence synthesis.

### 2.8. Effect Measures

Effect measures for all continuous outcomes—including total labeled motoneurons (LMN), labeled sensory neurons (LSN), total fibers (TF), fiber density (FD), and axon diameter (AD)—are reported as mean ± standard deviation (SD), where applicable. In studies using simultaneous retrograde tracing, the proportion of double- or triple-projecting neurons is expressed as a percentage (mean ± SD), where applicable. Sequential labeling studies contributed percentages of correctly directed motoneurons (mean ± SD), where applicable. Ratios derived from retrograde tracing and histomorphometric data are reported as relative numbers. As no standardized minimal clinically important difference thresholds exist for these animal models, all effect measures were calculated and reported using the original outcome-specific units to ensure consistency and comparability across included studies.

### 2.9. Synthesis Methods

Data synthesis was conducted in accordance with the PRISMA 2020 statement and the Synthesis Without Meta-analysis reporting framework [66] to ensure the clarity and reproducibility of our narrative approach. Studies were grouped into two primary intervention categories to evaluate the impact of different ArtNG structural designs on axonal misdirection by comparing them with direct ETE or BNG repair:

1. Unstructured ArtNGs (hollow NGCs without internal framework, further categorized by architecture to smooth and rough).
2. Structured ArtNGs (NSCs or filled NGCs, with internal framework, stratified by architecture to fibrous, multichannel, microporous).

**Figure 2:**
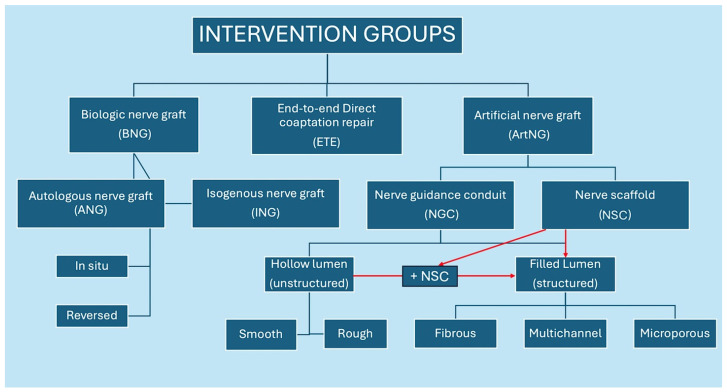
Nerve repair intervention groups and subgroups.

ArtNGs are further divided into NGCs and NSCs, while BNGs are divided into ANGs and INGs. A schematic presentation of the classification is provided in Figure 2 and detailed rationale in the following text.

The literature does not clearly distinguish between the terms “nerve guidance conduit (NGC)” and “nerve scaffold (NSC)”. While NSCs are often used as fillers within conduits, they are also commonly used independently as bridging materials. The main difference between these two is that NCSs do not provide a lumen for a nerve stump to be placed in during the repair, which is, according to the literature, a cornerstone characteristic of NGCs.

Furthermore, while the literature traditionally categorizes NGCs into hollow, porous, micropatterned, filled, and multichannel types, we found that most conduits incorporate characteristics spanning multiple categories. For example, electrospun nanofibrous conduits are often simultaneously porous and micropatterned, and may even be filled. This overlap complicated the subgroup classification, as the lack of standardized definitions made it difficult to clearly allocate interventions into discrete categories. When key information was missing, assumptions were not made conservatively and transparently. A challenging factor was that the authors of original studies often do not define their NGCs according to these commonly used structural classifications. In many cases, classification must be inferred indirectly, through a detailed assessment of the fabrication method, materials used, or, when available, an analysis of structural images and 3D scans. Without consistent reporting, structural categorization becomes subjective, introducing potential for bias or misinterpretation during synthesis.

Due to substantial heterogeneity across studies including differences in animal models, nerve types, gap lengths, follow-up durations, conduit architectures, and outcome measures a formal meta-analysis was not feasible. Instead, a directional effect assessment with vote counting was used to determine whether individual studies favored structured or unstructured ArtNGs for applicable outcome domain. Studies were further stratified into three comparison groups based on intervention characteristics and outcome assesment methods.

1. Direct ETE repair versus ArtNG repair.
2. ANG versus ArtNG repair assessed by single-dye tracing methods.
3. ANG versus ArtNG repair assessed by multiple-dye tracing methods.

Directional effect was assessed for each outcome domain by comparing ArtNGs with ANG or ETE. An upward arrow (↑) indicated a favorable outcome for ArtNGs, while a downward arrow (↓) indicated a favorable outcome for ANGs. Equality signs (=) denoted no clear difference, based on overlapping SDs, a mean difference of less than 10%, or non-significant *p*-values. The exception was for incorrect projecting LMNs, where lower values indicate better outcomes and ↑ was assigned when ArtNGs showed fewer misdirected motoneurons than ANGs.

When authors reported statistical tests or variance (SD/CI), classification followed the reported significance (α = 0.05). When variance was unavailable, a ±10% threshold versus the comparator was applied. Classifications based on thresholds are interpreted as directional trends, not statistical effects. Vote counting was then applied across these groups, providing the number of study arms that favored ArtNGs (↑), favored ANGs (↓), or showed no effect (=). This approach enabled identification of overall trends without requiring statistical pooling.

The heterogeneity was assessed by comparing distributions across subgroups using SPSS v.29 and visually through box plots. The certainty was qualitatively assessed using the GRADE domains (http://www.gradeworkinggroup.org/publications/JCE_series.htm, accessed on 30 April 2025):

- Risk of bias: based on SYRCLE tool
- Heterogeneity: noted when SD of the study means was large (>50% of mean)
- Indirectness: variation in species (rat vs. dog) or gap length
- Imprecision: small n of study arms per group (<5)
- No publication bias assessment was possible.

By adhering to both PRISMA and SWiM guidelines, this approach ensures the transparent, reproducible, and comprehensive synthesis of ArtNG structural features and their impact on axonal misdirection. Results were presented in structured summary tables, with bar graphs, and box plots used to visualize distributions across studies.

### 2.10. Automatization Tools and Language Based Models

ChatGPT (OpenAI, GPT-4) was used as a secondary tool to support data handling and manuscript preparation. Its use was limited to non-analytical, supportive functions, including (1) formatting tables and figures, (2) refining the clarity and consistency of written text, and (3) approximating numerical values from graphical data when not explicitly reported in the text. The tool was used to double-check manually extracted or formatted data, and all outputs were subsequently reviewed by human investigators to resolve any discrepancies and ensure accuracy. The inclusion of this additional verification layer enhanced the quality and consistency of the review but also extended the production timeline relative to a standard workflow. ChatGPT did not perform study selection, data extraction, or risk of bias assessment, all of which were conducted manually by independent reviewers.

## 3. Results

### 3.1. Study Selection

**Figure 3:**
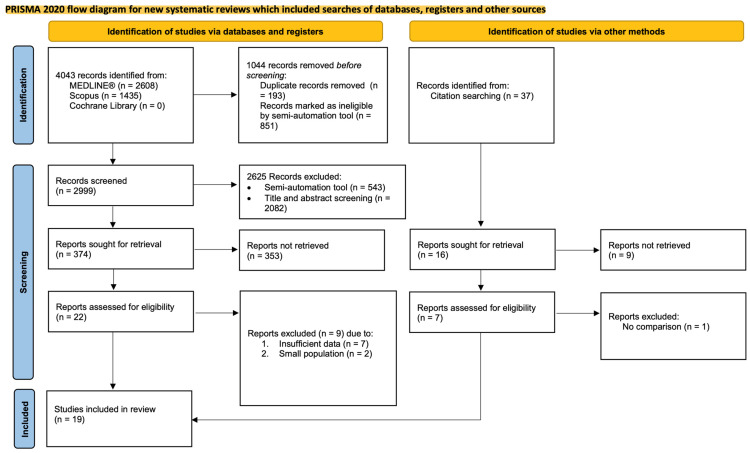
Flow diagram of article selection process designed according to PRISMA protocol [67].

The search strategy identified a total of 4043 articles (Figure 3). Before screening, 1044 records were removed (193 duplicates and 851 records marked as ineligible by a semi-automation tool) leaving 2999 records for title/abstract screening. During the title and abstract review, 2625 were excluded (543 by the semi-automation tool and human confirmation and 2082 after complete human screening) leaving 374 reports for retrieval. Upon retrieval, 22 records were selected for full-text review, out of which 7 were excluded due to insufficient data and two due to small population, leaving 13 studies for inclusion.

From 37 additional records identified from citation searching, 7 records were assessed for eligibility out of which 6 were included and one was excluded due to lack of comparison group.

### 3.2. Excluded Studies

Several promising studies were excluded due to insufficient motoneuron labeling data, small sample sizes, or incomplete reporting [68,69,70,71,72,73,74,75,76].

### 3.3. Study Characteristics

**Figure 4:**
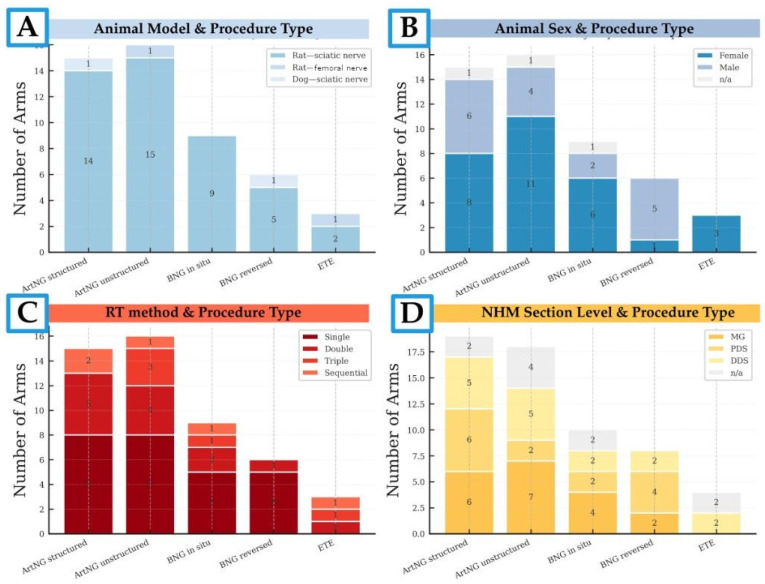
Composite bar charts illustrating the distribution of experimental groups characteristics animal model (**A**), sex (**B**), retrograde tracing method (**C**), and nerve histomorphometry section levels (**D**) by procedure type intervention types. ArtNG—artificial nerve graft, BNG—biologic nerve graft, ETE—end-to-end repair.

Of the 19 included studies comprising 49 experimental groups (Table 2), the rat sciatic nerve (SN) model was most frequently employed, represented in 17 studies encompassing 45 groups (Figure 4A). Sprague–Dawley rats were the predominant strain (n = 29 groups), followed by Lewis (n = 13) and Wistar (n = 5). Female animals were used in the majority of groups (n = 29), with the remainder including male (n = 17) or unspecified sexes (Figure 4B). The rat femoral nerve and dog sciatic nerve models were used in singular studies, involving 2 groups each.

Retrograde axonal labeling methods were variable across included studies (Figure 4C). Single-labeling was most common (26 groups), with 21 using Fluorogold (FG) and 3 using DiI on the SN, while two using FG on the tibial nerve. Double-labeling accounted for 13 groups—eleven employed Diamidino Yellow (DY) on the peroneal nerve and Fast Blue (FB) on the tibial nerve, while two used True Blue (TB) and DiI on femoral motor and sensory branches. All five triple-labeling studies utilized the DiI/FB/FG combination (DiI to the tibialis anterior branch, FB to the gastrocnemius branch, and FG to the peroneal motor branch). Out of five groups that used sequential labeling: three applied TB followed by FG on the tibial nerve, and two applied TB followed by DiI on femoral nerve branches.

Nerve histomorphometry was reported in 39 groups, including all single-labeled and all double-labeled groups (Figure 4D). Multiple anatomical levels were assessed in 26% of these groups, out of which 7 in single-labeling and 3 in double-labeling studies.

**Figure 5:**
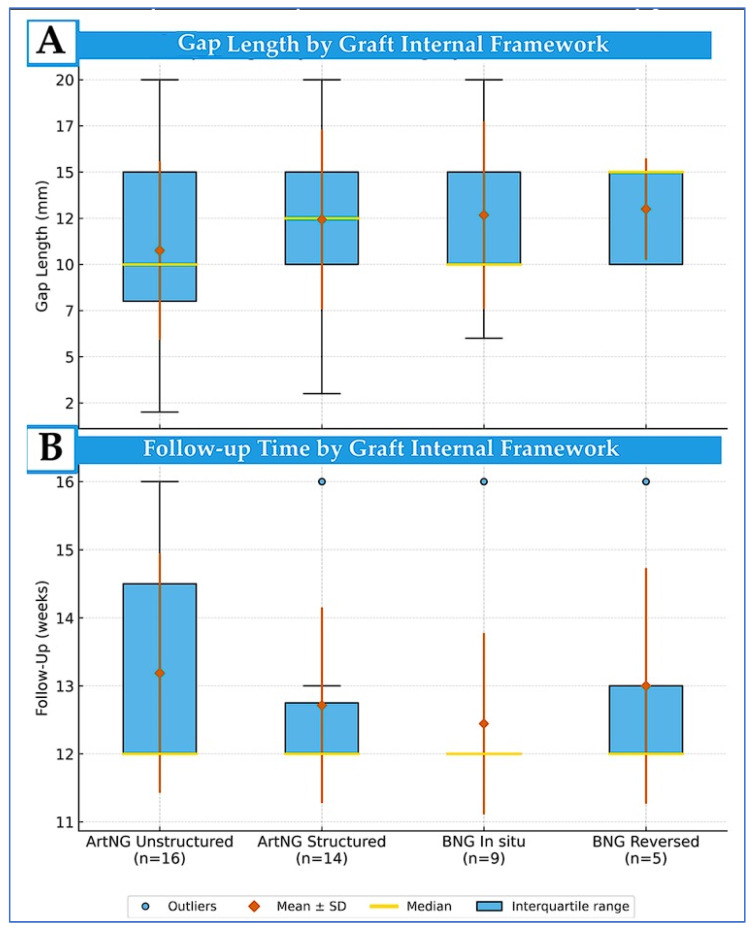
Distribution of nerve gap lengths (**A**) and follow-up durations (**B**) across four graft categories with exclusion of two groups involving dog model. Boxplots represent the interquartile range (IQR), with black whiskers extending to 1.5 × IQR and circles indicating outliers. Yellow-gold lines denote group medians; red-orange diamonds and bars represent group means ± standard deviations (SD). Number of experimental groups (n) are indicated beneath each category. ArtNG—artificial nerve graft. BNG—biologic nerve graft.

The distribution of gap lengths and follow-up durations across graft categories for 45 experimental groups is shown in Figure 5. The groups treated with ArtNGs exhibited the greatest variability due to inclusion of three studies with ArtNG application in short-gap models (2–5 mm) to compare with direct ETE repair. Follow-up duration showed less variability, with all graft categories displaying a median of 12 weeks and mean values between 12.5 and 13.0 weeks (IQRs: 12–13 to 12–14 weeks).

### 3.4. Risk of Bias in Studies

**Figure 6:**
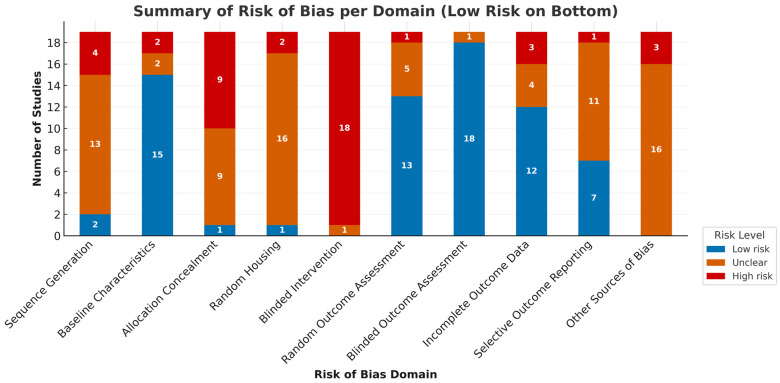
Risk of bias assessment summarized per domain.

The results of the risk of bias assessment are summarized in both tabular format and a color-coded heatmap (Table A3, Figure 6), presenting each domain’s risk judgment (low, unclear, or high) across all studies. The studies demonstrated a generally low methodological quality, with multiple domains at high or unclear risk.

Blinding of intervention was the most consistently problematic area, with 18 studies rated as high risk, suggesting that performance bias is a major concern across the evidence base. Random housing was also poorly addressed, with only three studies providing low-risk methods, while allocation concealment and sequence generation were frequently either inadequately described or at high risk, limiting confidence in the randomization process. In contrast, outcome-related domains such as blinded outcome assessment and random outcome assessment were more robust, with the majority of studies rated as low risk. However, incomplete outcome data and selective reporting varied, and many studies did not provide sufficient information to judge the presence of reporting bias or other potential sources of bias.

### 3.5. Results of Individual Studies

Individual study results are provided in Table S3. Key outcomes are presented below by domain and stratified by comparator group with directional effect and vote counting used to summarize study findings.

#### 3.5.1. LMN Counts

Total LMN counts were provided for all 19 studies. Results are summarized below according to comparator groups.

##### ArtNG vs. Direct ETE Repair

In all 3 studies comparing unstructured ArtNGs and direct ETE repair there were no significant differences in total LMN counts between the intervention and comparator group (Table 3). However, in 2/3 cases there was a Δ ≥ 10% mean difference, compared to ETE. There were no records comparing structured ArtNGs with direct ETE repair.

##### ArtNG vs. ANG Repair

Out of 28 ArtNG arms, 26 had study level ANG comparator, out of which 10 provided similar and 16 provided lower LMN counts compared to ANGs (Table 3). Of these 16, eight were significant at *p* < 0.01, four at *p* < 0.05, and four were reported as ≥10% decreases without *p*-values (Table 4 and Table 5.)

- Unstructured ArtNG arms, provided similar LMN counts in 2/11 and lower in 9/11 cases compared to ANGs.
- Structured ArtNG arms provided similar LMN counts in 8/15 and lower in 7/15 cases compared to ANGs. In 5/5 cases, longitudinal multichannel NSCs led to similar LMN counts, while randomized multichannel NSCs (2/2) and multichannel NGCs (3/3) led to lower LMN counts in all scenarios. Fibrous designs led to similar LMN counts in 3/5, and lower in 2/5 cases compared to ANGs.

#### 3.5.2. TLN Counts and Associated Ratios

Out of 13 ArtNG arms with TLN counts reported, 11 had study level ANG comparator. Out of 11, five were structured and 6 were unstructured.

- Structured ArtNG arms matched ANGs in TLN counts in 4/5, LSN counts in 3/5, and LMN/LSN ratio in 4/5 cases. LSN counts exceeded ANGs in one case. Regarding TLM/TF ratio, structured ArtNGs were similar to ANGs in one and superior in other case, out of two.
- Unstructured ArtNG arms matched ANGs in TLN counts in 1/6, LSN counts in 1/6 and LMN/LSN ratio in 3/5 cases. Regarding TLM/TF ration, unstructured ArtNGs were superior to ANGs in 4/5 and inferior in 1/5 cases.

#### 3.5.3. Motoneuron Projection Accuracy

Projection accuracy metrics were provided in 8 studies out of which 3 compared ArtNGs with direct ETE repair and 5 compared ArtNGs with ANGs (Table 5).

##### ArtNG vs. Direct ETE Repair

Across three short-gap neurorrhaphy comparisons, LMN survival was similar between ETE and conduits, but projection specificity diverged by design. In rats with hollow silicone NGCs, Bodine-Fowler et al. 1997 [77] found no difference in accurate projecting LMNs versus ETE (14 ± 5 vs. 19 ± 9). Valero Cabre et al. 2004 [79] also saw comparable LMN counts but a higher proportion of incorrect projections with silicone NGC than ETE (6.3% vs. 2.2%, *p* < 0.05), indicating worse specificity. In contrast, Yu et al. 2015 [85] (femoral nerve, 2-mm gap) reported better targeting with chitosan tubulation: incorrect projections were lower than ETE (74 ± 11 vs. 112 ± 13, *p* < 0.05) and accurate projections were higher (68.18 ± 2.04% vs. 55.89 ± 1.63%, *p* < 0.05).

##### ArtNG vs. ANG Repair

- Structured ArtNGs had lower percentage of incorrect projecting LMNs compared to ANGs in 4/7, similar in 2/7, and higher in 1/7 cases. Accurate projecting LMNs were reported only for the two arms and were similar to ANG in both cases.
- Unstructured ArtNGs provided similar percentage of incorrect projecting LMNs as ANGs in 3/5 and lower percentage in 2/5 cases. There were no data about accurate projecting LMNs across unstructured ArtNG arms.

#### 3.5.4. Other Outcomes

RN.AD/RN.FD ratios were reported in four studies (n = 13 arms). In short-gap models, structured conduits (0.06–0.13) aligned closely with autograft controls (0.05–0.13), whereas unstructured conduits produced higher ratios (0.15–0.44). In one long-gap study [87], both structured (9.85) and unstructured (20.79) exceeded in situ autograft values (9.76). No variance data were available, so these results are presented descriptively.

- Structured ArtNGs had similar AD/FD ratios in 2/5, lower in 2/5 and higher in 1/5 arms, when compared to ANGs.
- Unstructured ArtNGs had higher AD/FD ratios in 2/2 arms when compared to ANGs.

TF ratios between nerve segments were rarely reported, preventing any meaningful interpretation or directional analysis.

### 3.6. Synthesis of Results

Overall, structured ArtNGs demonstrated outcomes more closely aligned with ANG repair, while unstructured ArtNGs generally underperformed. Certainty of evidence across all domains was rated as very low, reflecting high risk of bias in included studies, small sample sizes, incomplete reporting of variance data, and indirectness inherent to animal models (Table 6).

- LMN counts: In 26 arms with ANG comparators, structured ArtNGs preserved LMN numbers in approximately half of cases (8/15), with longitudinal microchannel NSCs consistently matching ANGs (5/5). In contrast, unstructured ArtNGs provided similar LMN counts in only 2/11 arms, with 9/11 showing reductions.
- TLN counts: Structured ArtNGs preserved TLN in 4/5 arms, whereas unstructured guides showed preservation in just 1/6, with the remainder reporting decreases.
- LSN counts: Structured conduits preserved LSN in 3/5 arms and exceeded autografts in one arm, while unstructured conduits preserved LSN in only 1/6, with reductions in the others.
- LMN/LSN ratio: Structured conduits preserved motor–sensory balance in 4/5 arms, whereas unstructured guides were preserved in 3/5 but decreased in 2/5 arms.
- Projection accuracy: For incorrect projecting LMNs, structured conduits outperformed autografts in 4/7 arms, were similar in 2/7, and worse in 1/7; accurate projections were reported in only two structured arms, both comparable to ANG. Unstructured conduits reduced misdirected projections in 2/5 arms, matched in 3/5, and did not report accurate projection data.

### 3.7. Risk of Bias Due to Missing Values

Since only one outcome was consistently reported across all study groups, while the remaining outcomes did not exceed values of 50%, a pooled quantitative analysis could not be performed. The included studies were highly heterogeneous in terms of design, interventions, and reported endpoints. Furthermore, we judged the studies to carry a high risk of selective outcome reporting, as key functional and histological parameters were frequently missing or incompletely presented.

### 3.8. Certainty of Evidence

The overall certainty of evidence in this review is low to very low due to methodological limitations, small sample sizes, risk of bias, inconsistency, and imprecision across studies. While autografts consistently show benefits for motoneuron survival and regeneration, and structured NGCs may reduce axonal misdirection, the evidence is limited and uncertain, emphasizing trends rather than definitive conclusions.

## 4. Discussion

This systematic review provides the first comprehensive analysis of the structural impact of ArtNGs on axonal misdirection following peripheral nerve repair, focusing on studies implementing retrograde axonal tracing. Because of the lack of consensus on structural classification and the limited subset of features tested using, we adopted a pragmatic dichotomy: (1) structured (scaffolds and tubular conduits with internal framework and (2) unstructured (tubular conduits without internal framework). Despite this simplification, the evidence consistently indicates that structured ArtNGs more often performed similarly to or better than unstructured conduits, while ANGs remained superior across all domains. Because no single outcome providing assessment of axonal misdirection was reported in more than half of the experimental arms and overall certainty was very low, these observations should be interpreted as suggestive trends, not definitive conclusions.

The NGCs have been considered as significant factor that reduce axonal dispersion at the proximal stump [26] and provide initial directional alignment toward the distal stump [75,76], positioning this as a fundamental mechanism of nerve regeneration and recovery. However, no studies were identified that quantitatively assessed the impact of NGC structural design on proximal stump dispersion. In this review, only three included studies directly compared epineurial ETE with unstructured NGCs reporting similar, occasionally slightly higher LMN counts with NGCs. This may be due to the larger internal space of hollow conduits allowing more extensive axonal dispersion, whereas epineurial sutures may cause direct or indirect fiber trauma, with the interstump space further influenced by fibrin glue or fibrotic tissue formation.

Despite consistently reported equal LMN counts, the precision of reinnervation is generally reduced and, in some cases, increased. Reduced precision could be due to selective reinnervation driven by stronger distal cues, which in tubulation repair have more space and more fibers available to attract towards incorrect targets. While most of such studies used impermeable silicone hollow NGCs, in only case where ArtNGs achieved better reinnervation than direct ETE repair, the conduit was biodegradable and permeable. Such results should be reassessed as a potential consequence of a reduced selective reinnervation or diminished inhibition from sensory fibers toward motor fibers due to wall permeability and dilution of distal molecular factors. The dilution of distal molecular factors could be considered as contributing factor, regarding the satisfactory results in two studies when comparing non-soluble tube with epineurial ETE repair for distal coaptation site of autologous nerve graft [94,95].

This interpretation may be further supported by the findings of Liu et al. [89] the only included study to demonstrate superiority over ANG in reducing numbers of misdirected motoneurons. Notably, Liu et al. used structured fiber filled NGCs, which may have contributed to this outcome by reducing the influence of distal molecular cues through immediate physical guidance from its internal fibrous framework. This design provides a direct trajectory for axonal regrowth and limits the gap where distal misdirection could occur, particularly at a stage when distal molecular factors are already diluted.

The results of our studies aligns with the findings from earlier key experimental studies such as those by De Ruiter et al. [80] and Yao et al. [55], which showed that although structured conduits can mimic fascicular architecture, they still result in greater rates of double projections and lower reinnervation precision compared to ANGs. Studies using multichannel ArtNGs (Zhang et al., 2011 [83]; Bozkurt et al. 2016 [86]; Daly et al., 2012 [31]) showed an intermediate performance—better than hollow designs but not matching the fidelity of ANGs. Contrastingly, prior reviews often emphasized only histomorphometric or functional outcomes, without specifically assessing the reinnervation accuracy. This led to overly optimistic comparisons between NGCs and ANGs. Our review corrects this by integrating motoneuron labeling data and dispersion indices, revealing frequent misdirection in even structurally advanced NGCs.

The observed limitations of NGCs despite structural innovations reflect the complexity of axonal pathfinding, influenced not only by the luminal architecture but also by fascicular topography and intrinsic neuronal properties. Therefore, the current results underscore a growing consensus in the literature that physical guidance alone is insufficient for achieving target-specific reinnervation.

### 4.1. Limitations of the Evidence

The evidence included in this review is subject to several limitations that affect the overall certainty and generalizability of the findings. Most studies were preclinical and relied on small animal models (predominantly rat SN), limiting their translational relevance to human nerve repair. Sample sizes were frequently small, and many studies lacked key methodological safeguards such as randomization, blinding, or prospective protocol registration, increasing the risk of bias. Furthermore, significant heterogeneity was observed in conduit materials, fabrication methods, defect lengths, follow-up durations, and outcome metrics, complicating direct comparisons across studies. Reporting was often incomplete or selective—particularly regarding misdirection outcomes—leading to potential reporting bias. Finally, while retrograde tracing is the gold standard for assessing reinnervation accuracy, it remains a technically complex method with variable reproducibility, contributing to uncertainty in interpreting misdirection rates.

### 4.2. Limitations in the Review Process

Several limitations in the review processes may have influenced the findings of this systematic review. First, although a review protocol was written a priori, it was not registered in a public database, which may reduce transparency and increase the risk of post hoc changes to the eligibility criteria or analysis plans. Second, while data extraction and risk of bias assessment were performed in duplicate, with conflict resolution by a third reviewer, no attempts were made to contact the authors of studies for missing or unclear data, potentially leading to the exclusion of relevant information. Third, due to the methodological heterogeneity of the included studies, no meta-analysis was performed, limiting the ability to generate pooled effect estimates. Instead, a narrative synthesis following SWiM guidelines was used, which, although structured, remains inherently more subjective. Lastly, while automated tools were used to extract numerical data from figures, manual verification was not always possible, which may introduce minor inaccuracies. Together, these limitations may have introduced bias, reduced reproducibility, and affected the strength of conclusions.

### 4.3. Implications for Practice and Policy

The findings of this review suggest that while structured ArtNG designs, such as multichannel and fibrous tend to improve axonal guidance compared to unstructured hollow conduits, they do not yet match the reinnervation accuracy achieved with ANGs. Therefore, ArtNG should not currently be considered as replacements for autografts in clinical scenarios where precise functional recovery is critical, especially in large nerve gaps. However, structured designs may be considered as alternatives in select cases where autograft harvest is not feasible, particularly in small- to mid-sized nerve defects.

From a policy standpoint, the results highlight the need for stricter preclinical validation standards before novel ArtNG are translated into human use. Regulatory frameworks may benefit from incorporating axonal misdirection as a required outcome in preclinical conduit evaluation, rather than relying solely on general markers like fiber count or functional indices.

## 5. Future Perspectives

The following recommendations should be considered in future research regarding advancement of NGCs and NSCs:

1. Adopt standardized and comprehensive outcome reporting. Future studies should consistently include retrograde tracing alongside histomorphometry to assess both axonal dispersion and reinnervation accuracy.
2. Improve methodological rigor. Prospective protocol registration, randomization, blinding, and complete outcome reporting should become standard practice in preclinical nerve regeneration research.
3. Use larger animal models and longer follow-up periods. These steps would enhance the clinical relevance of findings and better simulate human nerve injury and repair conditions.
4. Isolate structural effects. Future experimental designs should separate the impact of the conduit structure from functional enhancements such as growth factors, stem cells, etc., to clarify the contribution of architecture alone.
5. Explore personalized, AI-assisted NGC design. Emerging imaging and 3D printing technologies, combined with machine learning, offer promising avenues for the development of patient-specific NGCs that replicate native fascicular anatomy.
6. Improve biomaterials, to provide microenvironment for axonal growth, by maintaining its physical structure, while being able to dissolve immediately when axons reach the distal stump.

Personalized 3D printed conduits with tubular branching patterns that match the intraneural fascicular topography of a patient sound promising as a future advance in NGC designing strategy.

Johnson et al. [96] demonstrated the usage of a 3D scanner for the intraoperative visualization of rat proximal and distal nerve stump morphology, computational design modeling, and the printing of tailored NGCs to match the stump fascicular distribution. Zhu et al. [97] developed a rapid continuous 3D printing platform for the fabrication of NGCs with various architectures and the adjustment of structural parameters to match the mechanical properties of the injury site.

In one study, Yao et al. demonstrated the usage of micro-magnetic resonance imaging (micro-MRI) for the scanning of fresh human nerve samples and their incorporation into computer software for 3D reconstruction [98]. In another study, Yao et al. used 3T MRI to scan patients’ healthy nerves at the contralateral side and designed a tailored nerve graft according to information from a previously formed database, in accordance with the specific injury situation [99].

Chang et al. [100] used serial histology sections, 3D reconstruction software, and computer-aided design to create a tailored NGC based on the fascicular topography of a rat sciatic nerve. Machine learning may be used in NGC computational design according to data from the created database [101,102].

Diffusion tensor imaging (DTI) plays a crucial role in nerve repair surgeries by providing pre-surgical insights into nerve pathways and the extent of damage [103]. Given its current role in assessing nerve integrity, DTI may also be utilized in the future for precise NGC placement, further improving targeted nerve regeneration and repair outcomes.

## 6. Summary

No current evidence clearly identifies the significant impact or superiority of any of the ArtNG structural designs, as studies vary widely in design, materials, and outcome reporting. Structured conduits with longitudinally oriented channels or fibers tend to improve axonal guidance over unstructured hollow designs, but still fall short of the precision achieved with ANGs. While small gap tubulation repair by ArtNGs may offer clinical potential, more standardized and rigorous research is necessary to fully understand potential of different structures and their role in the development of advanced, multifunctional, personaliyed, and AI-calibrated conduits with ultimate tendency to overcome ANGs

## Figures and Tables

**Table 1:** The eligibility criteria for study inclusion and exclusion based on PICO framework.

	Inclusion Criteria	Exclusion Criteria
Population	Mammalian in-vivo models of unilateral major peripheral nerve transection - sciatic nerve (SN) - tibial nerve (TN) - peroneal nerve (PN) - femoral nerve (FN) - median nerve (MN) - radial nerve (RN) - ulnar nerve (UN) ≥3 animals per group	Juvenile, elderly and models with genetically induced disease or deficiency Bilateral or multiple peripheral nerve injuries Proximal neural injuries - central motoneuron - cranial nerve - spinal cord - spinal root - spinal nerve injury - neural plexus injury Crush, compression, freeze, radiation or metabolic peripheral nerve injuries <3 animals per group Short-term follow up
Intervention	Immediate surgical repair Direct end-to-end (ETE) coaptation repair - epineurial suturing - non-suture techniques BNG (ANG or isogenous nerve graft (ING)) repair - Same nerve (in situ/reversed) - Distant nerve (straight/reversed) ArtNG (NGC or NSC) repair - Without structured architecture (smooth/rough/patterned) - With luminal architecture (multichannel/fibrous/spongious) - Porous/non-porous - Natural/Synthetic	Delayed surgical repair Interfascicular ETE coaptation repair End-to-side (ETS) coaptation repair Indirect nerve repair—nerve transfer Intentional misalignment at the coaptation site Combined procedure repair (BNG/ETE + ArtNG) Two stage procedures (two injuries/interventions at same nerve) Individualized/custom-made ArtNGs Non-linear ArtNGs (di-branched, multi-branched) Functionally enhanced ArtNGs - Incorporated or filled with cellular, molecular or physical factors that promote neural and/or glial proliferation - Fabricated with conductive or adhesive material Non neural biological grafts (Muscle, vein and other soft tissues) Acellular biological grafts (auto-, alo-, or xenografts)
Comparison	ETE vs. ArtNG (in small nerve gaps (≤5 mm)) BNG vs. ArtNG ArtNG vs. ArtNG (structurally different)	Studies without at least two included interventions to meet comparison criteria Studies involving ArtNGs with same structural features made of different material
Outcome	Retrograde axonal tracing (labeling) - Single labeling - Simultaneous labeling - Sequential labeling Nerve histomorphometry (optic, electron, fluorescent microscopy)	Qualitative presentation of study results - textual interpretation of statistical significance between the data - microscopic pictures with size bar - charts without error bar Single axonal labeling without nerve histomorphometry Nerve histomorphometry without axonal labeling

**Table 2:** Summarized characteristics of the included studies. The more detailed summary is provided in the Table S2. RT—retrograde tracing, NHM—nerve histomorphometry. SD Sprague–Dawley, W—Wistar, L—Lewis, B—Beagle. SN—sciatic nerve, FN—femoral nerve. ArtNG—artificial nerve graft, ETE—end-to-end coaptation repair, BNG—biologic nerve graft, ANG—autologous nerve graft. NGC—nerve guidance conduit, NSC nerve scaffold. SIL—silicone, PLGA—poly(lactic-co-glycolic acid), CS—chitosan, PGLA—poly(ε-caprolactone), CHIT—chitin, SF—silk fibroin. MG—mid-graft section, PDS—proximal to distal suture (coaptation) site, DDS—distal to distal suture (coaptation) site (s. distal nerve stump). NA—not applicable. NR—no reported.

Author, Year	Animal Species	Gap Length (mm)	Follow-Up (Weeks)	Repair Technique	Graft Type	Material	Internal Framework	Internal Achitecture	RT Technique	RT Cohort (n)	NHM Level	NHM Cohort (n)
Bodine-Fowler et al., 1997 [77]	SD female rat SN	NA	14	ETE	NA	NA	NA	NA	Sequential	9	NA	NA
		5	14	ArtNG	NGC	SIL	Unstructured	Smooth	Sequential	8	NA	NA
Valero Cabre et al., 2001 [78]	W female rat SN	8	12	BNG	ANG	-	In situ	-	Triple	6	NA	NA
		8	12	ArtNG	NGC	SIL	Unstructured	Smooth	Triple	6	NA	NA
		8	12	ArtNG	NGC	PLC	Unstructured	Smooth	Triple	8	NA	NA
Valero Cabre et al., 2004 [79]	W female rat SN	NA	12	ETE	NA	NA	NA	NA	Triple	9	NA	NA
		4	12	ArtNG	NGC	SIL	Unstructured	Smooth	Triple	13	NA	NA
De Ruiter et al., 2008 [80]	SD rat SN	10	12	BNG	ANG	NA	In situ	NA	Double	7	MG & DDS	6
		10	12	ArtNG	NGC	PLGA	Unstructured	Rough	Double	4	MG & DDS	4
		10	12	ArtNG	NGC	PLGA	Structured	Multichannel (7) *	Double	6	MG & DDS	2
Hu et al., 2009 [81]	SD male rat SN	15	12	ArtNG	NGC	SIL	Unstructured	Smooth	Single	3	DDS	3
		15	12	ArtNG	NSC	COL/CS	Structured	Multichannel (R) *	Single	3	DDS	5
		15	12	ArtNG	NSC	COL/CS **	Structured	Multichannel (L) *	Single	3	DDS	9
		15	12	BNG	ANG	-	Reversed	-	Single	3	DDS	9
Huang, 2010 [82]	SD male rat SN	15	12	ArtNG	NSC	CS	Structured	Multichannel (R) *	Single	5	PDS	10
		15	12	ArtNG	NSC	CS	Structured	Multichannel (L) *	Single	5	PDS	10
		15	12	BNG	ANG	NA	Reversed	NA	Single	5	PDS	10
Yao et al., 2010 [55]	L female rat SN	10	16	BNG	ANG	NA	In situ	NA	Double	6	MG	8
		10	16	ArtNG	NGC	COL	Unstructured	Rough	Double	6	MG	8
		10	16	ArtNG	NGC	COL	Structured	Multichannel (2) *	Double	6	MG	8
		10	16	ArtNG	NGC	COL	Structured	Multichannel (4) *	Double	6	MG	8
Zhang, 2011 [83]	SD male rat SN	15	12	ArtNG	NSC	COL	Structured	Multichannel (L) *	Single	6	MG & PDS	6
		15	12	BNG	ANG	NA	Reversed	NA	Single	6	MG & PDS	6
Daly, 2012 [31]	L female rat SN L female rat SN L female rat SN L female rat SN	10	13	BNG	ANG	NA	Reversed	NA	Double	6	MG	7
		10	13	ArtNG	NGC	COL	Structured	Fibrous (S) *	Double	6	MG	8
		10	13	ArtNG	NGC	COL	Structured	Fibrous (U) *	Double	6	MG	8
		10	13	ArtNG	NGC	COL	Unstructured	Rough	Double	6	MG	8
Jiang, 2012 [84]	SD female rat SN	15	16	ArtNG	NGC	PCL	Unstructured	Rough (M) *	Single	8	MG	8
	SD female rat SN	15	16	ArtNG	NGC	PCL	Unstructured	Rough (N) *	Single	9	MG	9
Yu, 2015 [85]	SD female rat SN	-	12	ETE	NA	NA	NA	NA	Double	10	DDS	6
	SD female rat SN	2	12	ArtNG	NGC	CHIT	Unstructured	Smooth	Double	10	DDS	6
Bozkurt, 2016 [86]	L female rat SN	20	12	ArtNG	NSC	COL	Structured	Multichannel (L) *	Single	4	PDS & DDS	7
	L female rat SN	20	12	BNG	ANG	-	In situ	NA	Single	5	PDS & DDS	6
Van Neerven et al., 2017 [87]	L female rat SN	20	12	ArtNG	NGC	COL	Structured	Multichannel (L) *	Single	4	PDS & DDS	6
	L female rat SN	20	12	ArtNG	NGC	COL	Unstructured	Rough	Single	4	PDS & DDS	8
	L female rat SN	20	12	BNG	ANG	NA	In situ	NA	Single	5	PDS & DDS	5
Zhu, 2017 [88]	SD male rat SN	15	12	ArtNG	NGC	PCL	Unstructured	Rough	Single	9	MG	3
	SD male rat SN	15	12	BNG	ANG	NA	In situ	NA	Single	9	MG	3
Liu, 2018 [89]	SD female rat SN	6	12	BNG	ANG	NA	In situ	NA	Sequential	6	NA	NA
	SD female rat SN	3	12	ArtNG	NGC	PLGA-CS ***	Structured	Fibrous	Sequential	6	NA	NA
	SD female rat SN	6	12	ArtNG	NGC	PLGA-CS ***	Structured	Fibrous	Sequential	6	NA	NA
Xue, 2018 [90]	B male dog SN	30	52	ArtNG	NGC	SF	Structured	Fibrous	Single	3	PDS	6
		30	52	BNG	ANG	NA	Reversed	NA	Single	3	PDS	6
Zhu, 2020 [91]	SD male rat SN	15	12	ArtNG	NGC	PCL	Unstructured	Rough	Single	6	MG	6
		15	12	BNG	ANG	NA	In situ	NA	Single	6	MG	6
Lu, 2021 [92]	SD female rat SN	10	12	ArtNG	NGC	CHIT	Unstructured	Smooth	Single	4	DDS	5
		10	12	BNG	ANG	NA	In situ	NA	Single	4	DDS	5
Yang, 2023 [93]	SD male rat SN	10	16	ArtNG	NGC	CS	Unstructured	Smooth	Single	3	PDS	3
		10	16	BNG	ANG	NA	Reversed	NA	Single	3	PDS	3

* Braces contain additional structural information. (2), (4), (7)—number of channels in multichannel NGCs. (S)—structured luminal fibers, (N)—nonstructured luminal fibers, (M)—microfibrous NGC, (N)—nanofibrous NGC., (R)—randomized multichannel NSC, (L)—longitudinal multichannel NSC. ** Two materials reported with (/) in between—both used as components for fabrication of NGC wall. *** Two materials reported with (-) in between—first used for fabrication of NGC wall, second used for fabrication of luminal fibers.

**Table 3:** Summarized results of studies reporting on direct ETE vs. small gap ArtNG repair. Arrows indicate the direction of effect based on vote counting across study arms: ↑ denotes outcomes favoring artificial nerve grafts (ArtNGs), ↓ denotes outcomes favoring direct end-to-end (ETE) repair, and = denotes no difference between the groups.

Study ID	Animal Model	Repair Technique Characteristics			LMN	Incorrect Projecting LMNs (%)		Accurate Projecting LMN (%)
Bodine-Fowler et al., 1997 [77]	Rat (SD), SN,	ETE	112 ± 38	REF	NA	NA	19 ± 9	REF
	Rat (SD), SN, 5 mm	ArtNG, NGC, SIL, Unstructured, Smooth	133 ± 41	=	NA	NA	14 ± 5	=
Key finding: Despite similar LMN survival, there were no significant differences between the amount of accurate projecting LMNs between the hollow SIL NGC and direct ETE repair (14 ± 5 vs. 19 ± 9).								
Valero Cabre et al., 2004 [79]	Rat (W), SN,	ETE	1175 ± 106	REF	2.2	REF	NA	NA
	Rat (W), SN, 4 mm	ArtNG, NGC, SIL, Unstructured, Smooth	1250 ± 182	=	6.3	↑ (*p* < 0.05)	NA	NA
Key finding: Despite similar LMN survival, the percentage of incorrect projections was significantly higher in NGC (6%) compared to ETE repair (2.2%), with *p* < 0.05.								
Yu et al., 2015 [85]	Rat (SD), FN,	ETE	334 ± 38	REF	112 ± 13	REF	55.89 ± 1.63	REF
	Rat (SD), FN, 2 mm	ArtNG, NGC, CHIT, Unstructured, Smooth	326 ± 42	=	74 ± 11	↓ (*p* < 0.05)	68.18 ± 2.04	↑ (*p* < 0.05)
Key finding: Despite similar LMN survival, the percentage of incorrect projections (74 ± 11%) was significantly lower in NGC compared to ETE repair (112 ± 13) (*p* < 0.05). This observation is reflected in a significant difference in the specificity of motor axon regeneration between the two groups (small gap tubulization group: 68.18 ± 2.04% vs. epineurial neurorrhaphy group: 55.89 ± 1.63%, *p* < 0.05)								

**Table 4:** Summarized results of studies analyzed by the single retrograde tracing methods. Arrows indicate the direction of effect based on vote counting across study arms: ↑ denotes outcomes favoring artificial nerve grafts (ArtNGs), ↓ denotes outcomes favoring autologous nerve grafts (ANGs), and = denotes no difference between groups (no clear advantage for either ArtNGs or ANGs). DDS.AD—Axon diameter distally to the distal coaptation (suture) site, DDS.FD—Fiber density distally to the distal coaptation (suture) site.

Study ID	Animal Model	Repair Technique Characteristics	LMN		LSN		TLN		LMN/LSN		TLN/RN.TF		TLN/DDS	
Hu et al. [81] 2009	Rat (SD), SN, 15 mm	ArtNG, NGC, SIL, Unstructured, Smooth	230 ± 30	↓ (*p* < 0.05)	260 ± 50	↓ (*p* < 0.05)	490 ± 58.3	↓ (Δ ≥ 10%)	0.88 ± 0.21	= (overlap)	NR	NA	0.09	↑
		ArtNG, NSC, COL-CS*, Structured, Multichannel (R)	550 ± 85	↓ (*p* < 0.05)	480 ± 60	↓ (*p* < 0.05)	1030 ± 104.0	↓ (Δ ≥ 10%)	1.15 ± 0.23	= (overlap)	NR	NA	0.10	↑
		ArtNG, NSC, COL-CS*, Structured, Multichannel (L)	700 ± 90	= (*p* > 0.05)	720 ± 70	= (*p* > 0.05)	1420 ± 114.0	= (overlap)	0.97 ± 0.16	= (overlap)	NR	NA	0.06	↓
		BNG, ANG, Reversed	800 ± 80	REF	820 ± 80	REF	1620 ± 113.1	REF	0.98 ± 0.14	REF	NR	NA	0.07	REF
Key findings: There was no significant difference in LMN and LSN counts between longitudinal multichannel NSCs and reversed ANGs (*p* > 0.05), while significantly lower in two other ArtNg Groups (*p* < 0.05).														
Huang et al., 2010 [82]	Rat (SD), SN, 15 mm	ArtNG, NSC, CS, Structured, Multichannel (R)	390 ± 12	↓ (*p* < 0.05)	NR	NA	NR	NA	NR	NA	NR	NA	NR	NA
		ArtNG, NSC, CS, Structured, Multichannel (L)	520 ± 12	= (*p* > 0.05)	NR	NA	NR	NA	NR	NA	NR	NA	NR	NA
		BNG, ANG, Reversed	530 ± 12	REF	NR	NA	NR	NA	NR	NA	NR	NA	NR	NA
Key findings: There was a significant difference in LMN counts between longitudinal and randomized multichannel NSCs (*p* < 0.05).														
Zhang et al., 2011 [83]	Rat (SD), SN, 15 mm	ArtNG, NSC, COL, Structured, Multichannel (L)	520 ± 20	= (*p* > 0.05)	580 ± 20	= (*p* > 0.05)	1100 ± 28.3	= (Δ < 10%)	0.90 ± 0.05	= (overlap)	0.06	=	NR	NA
		BNG, ANG, Reversed	550 ± 20	REF	650 ± 20	REF	1200 ± 28.3	REF	0.85 ± 0.04	REF	0.06	REF	NR	NA
Key findings: There was no significant difference in LMN and LSN counts between longitudinal multichannel NSCs and reversed ANG (*p* > 0.05).														
Jiang et al., 2012 [84]	Rat (SD), SN, 15 mm	ArtNG, NGC, PCL, Unstructured, Rough Microfibrous	270 ± 141	NA	980 ± 849	NA	1250 ± 860	NA	0.28 ± 0.28	NA	0.39	NA	NR	NA
		ArtNG, NGC, PCL, Unstructured, Rough Nanofibrous	360 ± 180	NA	1930 ± 2130	NA	2290 ± 2138	NA	0.19 ± 0.23	NA	0.34	NA	NR	NA
Key findings: There was no significant difference in LMN counts, while there were significantly higher LSN and TF counts in nanofibrous compared to microfibrous NGCs (*p* < 0.05).														
Bozkurt et al., 2016 [86]	Rat (L), SN, 20 mm	ArtNG, NSC, COL, Structured, Multichannel (L)	454 ± 358	= (overlap)	760 ± 174	= (overlap)	1214 ± 398	= (overlap)	0.60 ± 0.49	= (overlap)	NR	NA	NR	NA
		BNG, ANG, In situ	650 ± 168	REF	900 ± 179	REF	1550 ± 245	REF	0.72 ± 0.24	REF	NR	NA	NR	NA
Key findings: No reported significant difference in outcomes between in situ ANG and longitudinal multichannel NCSs.														
Van Neerven et al., 2017 [87]	Rat (L), SN, 20 mm	ArtNG, NGC, COL, Structured, Multichannel (L)	290 ± 184	= (overlap)	1424 ± 338	↑ (Δ ≥ 10%)	1714 ± 385	= (overlap)	0.20 ± 0.14	↓ (Δ ≥ 10%)	0.44	↑	0.8	=
		ArtNG, NGC, COL, Unstructured, Rough	206 ± 180	= (overlap)	1126 ± 522	= (overlap)	1332 ± 552	= (overlap)	0.18 ± 0.18	↓ (Δ ≥ 10%)	0.58	↑	0.8	=
		BNG, ANG, In situ	456 ± 94	REF	785 ± 192	REF	1241 ± 214	REF	0.58 ± 0.19	REF	0.08	REF	0.1	REF
Key findings: Structured ArtNG resulted in significantly higher PDS.FD compared to unstructured ArtNG (*p* < 0.05), and higher DDS.AD and DDS.FD compared to BNGs (*p* < 0.01).														
Zhu et al., 2017 [88]	Rat (SD), SN, 15 mm	ArtNG, NGC, PCL, Unstructured, Rough	490 ± 40	↓ (Δ ≥ 10%)	570 ± 50	↓ (Δ ≥ 10%)	1060 ± 64.0	↓ (Δ ≥ 10%)	0.86 ± 0.10	= (overlap)	0.44	↑	NR	NA
		BNG, ANG, In situ	580 ± 35	REF	680 ± 40	REF	1260 ± 53.2	REF	0.85 ± 0.07	REF	0.12	REF	NR	NA
Key findings: No reported significant difference in outcomes between in situ ANG and unstructured rough NGC.														
Xue et al., 2018 [90]	Dog (B), SN, 30 mm	ArtNG, NGC, SF, Structured, Fibrous	1700 ± 300	= (overlap)	NR	NA	NR	NA	NR	NA	NR	NA	NR	NA
		BNG, ANG, Reversed	1800 ± 200	REF	NR	NA	NR	NA	NR	NA	NR	NA	NR	NA
Key findings: No reported significant difference in outcomes between reversed ANG and structured fibrous NSC.														
Zhu et al., 2020 [91]	Rat (SD), SN, 15 mm	ArtNG, NGC, PCL, Unstructured, Rough	500 ± 30	↓ (*p* < 0.01)	580 ± 30	↓ (Δ ≥ 10%)	1080 ± 42.4	↓ (Δ ≥ 10%)	0.86 ± 0.07	= (Overlap)	0.09	↑	NR	NA
		BNG, ANG, In situ	635 ± 69	REF	705 ± 56	REF	1340 ± 88.9	REF	0.90 ± 0.12	REF	0.07	REF	NR	NA
Key findings: ANGs provided significantly higher LMN (*p* < 0.01) and TF (*p* < 0.05) counts compared to unstructured ArtNGs.														
Lu et al., 2021 [92]	Rat (SD), SN, 10 mm	ArtNG, NGC, CHIT, Unstructured, Smooth	195 ± 10	↓ (*p* < 0.01)	300 ± 10	↓ (*p* < 0.01)	495 ± 14.1	↓ (Δ ≥ 10%)	0.65 ± 0.04	↓ (Δ ≥ 10%)	NR	NA	NR	NA
		BNG, ANG, In situ	420 ± 20	REF	570 ± 10	REF	990 ± 22.4	REF	0.74 ± 0.04	REF	NR	NA	NR	NA
Key findings: ANGs provided significantly higher LMN, LSN, and DDS.FD (*p* < 0.01) counts compared to unstructured ArtNGs.														
Yang et al., 2023 [93]	Rat (SD), SN, 10 mm	ArtNG, NGC, CS, Unstructured, Smooth	95 ± 10	↓ (*p* < 0.01)	310 ± 20	↓ (Δ ≥ 10%)	405 ± 22.4	↓ (Δ ≥ 10%)	0.31 ± 0.04	↓ (Δ ≥ 10%)	0.04	↓	NR	NA
		BNG, ANG, Reversed	140 ± 5	REF	375 ± 5	REF	515 ± 7.1	REF	0.37 ± 0.01	REF	0.11	REF	NR	NA
Key findings: ANGs provided significantly higher LMN (*p* < 0.01) counts compared to unstructured ArtNGs.														

**Table 5:** Summarized results of studies analyzed by the double, triple and sequential retrograde tracing methods. Arrows indicate the direction of effect based on vote counting across study arms: ↑ denotes outcomes favoring artificial nerve grafts (ArtNGs), ↓ denotes outcomes favoring autologous nerve grafts (ANGs), and = denotes no difference between groups (no clear advantage for either ArtNGs or ANGs.

Study ID	Animal Model	Repair Technique Characteristics	LMN		Incorrect Projecting LMNs (%)		Accurate Projecting LMNs (%)	
Valero Cabre et al., 2001 [78]	Rat (W), SN, 8 mm	BNG, ANG, In situ	1186 ± 56	REF	5.8 ± 0.6	REF	NA	NA
		ArtNG, NGC, SIL, Unstructured, Smooth	935 ± 213	= (Overlap)	10 ± 0.1	↓ (*p* < 0.05)	NA	NA
		ArtNG, NGC, PLC, Unstructured, Smooth	802 ± 101	↓ (*p* < 0.05)	6.0 ± 1.6	= (Δ < 10%)	NA	NA
Key finding: The percentage of double projecting neurons was significantly higher with hollow SIL NGC (10.1%) compared to autograft (5.8%) and PLC NGC (6.0%), with (*p* < 0.05).								
De Ruiter et al., 2008 [80]	Rat (SD), SN, 10 mm	BNG, ANG, In situ	1140 ± 179	REF	5.9 ± 2.9	REF	NA	NA
		ArtNG, NGC, PLGA, Unstructured, Rough	406 ± 156	↓ (Δ ≥ 10%)	21.4 ± 4.9	↓ (*p* < 0.01)	NA	NA
		ArtNG, NGC, PLGA, Structured, Multichannel (7)	448 ± 108	↓ (Δ ≥ 10%)	16.9 ± 6.0	↓ (Δ ≥ 10%)	NA	NA
Key finding: The percentage of double projecting neurons was 21.4% compared to autograft repair (5.9%) being significantly lower with a p value less than 0.01								
Yao et al., 2010 [55]	Rat (L), SN, 10 mm	BNG, ANG, In situ	1000 ± 167	REF	4.5 ± 2.4	REF	NA	NA
		ArtNG, NGC, COL, Unstructured, Rough	270 ± 64	↓ (*p* < 0.01)	7.1 ± 2.7	= (Overlap)	NA	NA
		ArtNG, NGC, COL, Structured, Multichannel (2)	245 ± 50	↓ (*p* < 0.01)	2.7 ± 2.9	= (Overlap)	NA	NA
		ArtNG, NGC, COL, Structured, Multichannel (4)	325 ± 75	↓ (*p* < 0.01)	2.4 ± 1.5	= (Overlap)	NA	NA
Key finding; Percentage of LMNs with double projections in hollow COL NGC (7.1% ± 2.7%), was significantly higher when compared with treated 2-channel and 4-channel conduits (*p* < 0.05).								
Daly et al., 2012 [31]	Rat (L), SN, 10 mm	BNG, ANG, Reversed	980 ± 269	REF	17.83 ± 13.57	REF	NA	NA
		ArtNG, NGC, COL, Structured, Fibrous (S)	880 ± 184	= (Overlap)	0.84 ± 1.19	↑ (*p* > 0.05)	NA	NA
		ArtNG, NGC, COL, Structured, Fibrous (U)	730 ± 171	= (Overlap)	2.42 ± 2.33	↑ (*p* > 0.05)	NA	NA
		ArtNG, NGC, COL, Unstructured, Rough	260 ± 147	↓ (Δ ≥ 10%)	6.8 ± 1.1	↑ (Δ ≥ 10%)	NA	NA
Key finding: Structured fibers and unstructured fibers showed significantly lower misdirection rates (Structured: 0.84% ± 1.19%, Unstructured: 2.42% ± 2.33%) compared to autograft (17.83% ± 13.57%) (*p* < 0.05).								
Liu et al., 2018 [89]	Rat (SD), SN, 6 mm	BNG, ANG, In situ	2144 ± 205	REF	30.50 ± 3.25	REF	83.98 ± 5.41	REF
		ArtNG, NGC, PLGA/CS, Structured, Fibrous	1886 ± 292	↓ (*p* < 0.01)	20.33 ± 8.10	↑ (*p* < 0.05)	83.43 ± 9.48	= (Overlap)
		ArtNG, NGC, PLGA/CS, Structured, Fibrous	1544 ± 228	↓ (*p* < 0.01)	19.45 ± 9.21	↑ (*p* < 0.05)	83.98 ± 6.76	= (Overlap)
Key finding: The tubulation repair with a 3 mm chitosan/PGLA conduit significantly reduced motor axon misdirection compared to autograft repair, despite slightly lower total motor neuron regeneration.								

**Table 6:** Summary of outcomes comparing structured versus unstructured ArtNGs in preclinical retrograde tracing studies. Data are reported as the number of experimental arms showing similar, lower, or higher values relative to ANGs. Certainty of evidence is rated as very low due to preclinical design, high risk of bias, and imprecision. The table was generated using OpenAI ChatGPT 4.

Outcome	Structured ArtNGs	Unstructured ArtNGs	No. of Arms (Studies)	Certainty of Evidence	Plain Language Summary
LMN counts	8/15 similar, 7/15 lower; microchannel 5/5 preserved; microporous & multichannel 100% lower	2/11 similar, 9/11 lower	26 arms (19 studies)	⬤◯◯◯ Very low	Structured ArtNGs often preserved LMNs; unstructured conduits mostly reduced LMNs vs. ANG
TLN counts	4/5 similar	1/6 similar, 5/6 lower	11 arms (13 studies)	⬤◯◯◯ Very low	Structured ArtNGs generally preserved TLN; unstructured conduits mostly reduced TLN
LSN counts	3/5 similar, 1/5 higher	1/6 similar, 5/6 lower	11 arms (13 studies)	⬤◯◯◯ Very low	Structured ArtNG occasionally increased LSN; unstructured conduits often reduced LSN
LMN/LSN ratio	4/5 similar	3/5 similar, 2/5 lower	10 arms (13 studies)	⬤◯◯◯ Very low	Structured conduits more often preserved motor–sensory specificity, while unstructured more often worsened it
Innervation accuracy—Incorrect projecting LMNs	4/7 lower, 2/7 similar, 1/7 higher	2/5 lower, 3/5 similar	12 arms (8 studies)	⬤◯◯◯ Very low	Structured conduits more often reduced misdirection; unstructured presented with mixed results
Innervation accuracy—Accurate projecting LMNs	2/7 similar	0/5 reported	2 arms (2 studies)	⬤◯◯◯ Very low	Sparse reporting was present—structured conduits matched ANG in two arms

## Data Availability

Currently, all data are obtainable by contacting the authors.

## Supplementary Materials

The following supporting information can be downloaded at: https://www.mdpi.com/article/10.3390/bioengineering13020220/s1, Table S1: Final PubMed Search Query; Table S2: AI-generated translation of PubMed search code query for EBSCO discovery service; Table S3: Final Data Supositoria.

## Appendix A

**Table A1:** Search terms formed within PICO framework.

PICO		Key Words	Search Terms
**Population**	Animals	Animals Mammals Models	(’’Animals’’[MH]) OR (‘’Models, Animal’’[MH]) OR (‘’Animal Experimentation’’[MH]) OR (rodent[Title/Abstract]) OR (rat[Title/Abstract]) OR (mouse[Title/Abstract]) OR (mice[Title/Abstract]) OR (hamster[Title/Abstract]) OR (monkey[Title/Abstract]) OR (’’guinea pig’’[Title/Abstract]) OR (pig[Title/Abstract]) OR (rabbit[Title/Abstract]) OR (cat[Title/Abstract]) OR (dog[Title/Abstract]) OR (sheep[Title/Abstract])
	Peripheral Nerve Injury	Nerve Injury Axotomy Nerve Gap Nerve Transection	(‘’Peripheral Nerve Injuries’’[MH]) OR (‘’Axotomy’’[MH]) OR (‘’Nerve Crush’’[MH]) OR (’’Nerve Regeneration’’[MH]) OR (Nerve Injury Model’’[Title/Abstract]) OR (’’Nerve Crush’’[Title/Abstract]) OR (’’Nerve Crush Model’’[Title/Abstract]) OR (’’Axonotmesis’’[Title/Abstract]) OR (’’Neurotmesis’’[Title/Abstract]) OR (’’Nerve Transection’’[Title/Abstract]) OR (’’Nerve Transection Model’’[Title/Abstract]) OR (’’Nerve Cut’’[Title/Abstract]) OR (’’Nerve Gap’’[Title/Abstract]) OR (’’Nerve Defect’’[Title/Abstract])
**Intervention**	Nerve Guidance Conduit	Nerve Guide Nerve Conduit Artificial Nerve Graft Nerve Scaffold	(‘’Tissue Scaffolds’’[MeSH terms]) OR (‘’Nerve Guidance Conduits’’[Title/Abstract]) OR (‘’Nerve Guidance Channels’’[Title/Abstract]) OR (NGCs[Title/Abstract]) OR (‘’Nerve Conduits’’[Title/Abstract]) OR (‘’Neural Conduits’’[Title/Abstract]) OR (‘’Neural Tubes’’[Title/Abstract]) OR (‘’Nerve Tubes’’[Title/Abstract]) OR (‘’Nerve Scaffold’’[Title/Abstract]) OR (‘’Neural Scaffold’’[Title/Abstract]) OR (‘’Artificial Nerve Graft’’[Title/Abstract]) OR (‘’Artificial Nerve Conduit’’[Title/Abstract]) OR (‘’Fabricated Nerve Graft’’[Title/Abstract]) OR (‘’Synthetic Nerve Graft’’[Title/Abstract]) OR (‘’Nerve Guide’’[Title/Abstract]) OR (‘’Nerve Connector’’[Title/Abstract])
	Structural design	Hollow Porous Grooved Nanofiber Nanosponge Multichannel	(‘’Biomimetics’’[MeSH terms]) OR (‘’Nanostructures’’[MeSH terms]) OR (’’Tissue’’Guided Tissue Regeneration’’[MeSH terms]) OR (Structure[Title/Abstract]) OR (Design[Title/Abstract]) OR (Property[Title/Abstract]) OR (‘’Structural Design Strategy’’[Title/Abstract]) OR (Architecture[Title/Abstract]) OR (Microarchitecture[Title/Abstract]) OR (Microstructure[Title/Abstract]) OR (Superstructure[Title/Abstract]) OR (Topography[Title/Abstract]) OR (‘’Lumen Topography’’[Title/Abstract]) OR (‘’Lumen Feature’’[Title/Abstract]) OR (‘’Lumen Surface Feature’’[Title/Abstract]) OR (‘’Structural Feature’’[Title/Abstract]) OR (’’Mechanical Cues’’[Title/Abstract]) OR (’’Topographic Cues’’[Title/Abstract]) OR (Topographic alignment[Title/Abstract]) OR (Porous[Title/Abstract]) OR (Nanopores[Title/Abstract]) (Grooved[Title/Abstract]) OR (Microgrooved[Title/Abstract]) OR (Filled[Title/Abstract]) OR (’’Luminal Fillers’’[Title/Abstract]) OR (Nanofiber[Title/Abstract]) OR (‘’Filament fiber’’[Title/Abstract]) OR (Hydrogel[Title/Abstract]) OR (Nanosponge[Title/Abstract]) OR (Micropore[Title/Abstract]) OR (Multichannel[Title/Abstract]) OR (Microchannel[Title/Abstract]) OR (Multilumen[Title/Abstract]) OR (‘’Single Channel’’[Title/Abstract]) OR (‘’Single Lumen’’[Title/Abstract]) OR (‘’Single Hollow’’[Title/Abstract]) OR (Multibranched[Title/Abstract]) OR (Branched[Title/Abstract]) OR (Micropatterned[Title/Abstract]) OR (Bibranched[Title/Abstract]) OR (Tubular[Title/Abstract]) OR (Spiral[Title/Abstract]) OR (’’Channel Orientation’’[Title/Abstract]) OR (’’Channel Number’’[Title/Abstract]) OR (’’Pores’’[Title/Abstract]) OR (’’Holes’’[Title/Abstract])
**Comparison**	Surgical nerve repair	Nerve repair End-to-end repair Autograft	(Neurotization[MeSH terms]) OR (‘’Suture Techniques’’[MeSH terms]) OR (’’Sutureless Surgical Procedures’’[MeSH terms]) OR (Autografts[MeSH terms]) OR (Neurotization[Title/Abstract]) OR (Autografts[Title/Abstract]) OR (’’Nerve Reconstruction’’[Title/Abstract]) OR (’’End-to-end suture’’[Title/Abstract]) OR (’’epineurial suture’’[Title/Abstract]) OR (’’Graft Repair’’[Title/Abstract]) OR (’’Nerve Repair’’[Title/Abstract]) OR (’’Direct Nerve Repair’’[Title/Abstract]) OR (’’Direct Nerve Suture’’[Title/Abstract]) OR (Microsuture[Title/Abstract]) OR (’’Nerve Surgery’’[Title/Abstract])
**Outcome**	Axonal misdirection	Axonal guidance Axonal pathfinding Axonal sprouting Axonal dispersion Axonal alignment Reinnervation mismatch	(Neurogenesis[MeSH terms]) OR (’’Neuronal Outgrowth’’[MeSH terms]) OR (’’Axon Pathfinding’’[MeSH terms]) OR (’’Axon Fasciculation’’[MeSH terms]) OR (’’Axon Guidance’’[MeSH terms]) OR (’’Axon* Bundling’’[Title/Abstract) OR (’’Neurite Fasciculation’’[Title/Abstract]) OR (’’Neural Pathfinding’’[MeSH terms]) OR (’’Neural Guidance’’[Title/Abstract]) OR (’’Axon* Pruning’’[Title/Abstract]) OR (’’Neurite Pruning’’[Title/Abstract]) OR (’’Neurite Arborization’’[Title/Abstract]) OR (’’Axon* Arborization’’[Title/Abstract]) OR (’’Synaptic Pruning’’[Title/Abstract]) OR (‘’Axon* Pathway’’[Title/Abstract]) OR (‘’Axon* Misdirection’’[Title/Abstract]) OR (‘’Axon* Direction’’[Title/Abstract]) OR (‘’Axon* Alignment’’[Title/Abstract]) OR (‘’Axon* Misalignment’’[Title/Abstract]) OR (‘’Axon* Orientation’’[Title/Abstract]) OR (‘’Axon* Navigation’’[Title/Abstract]) OR (‘’Axon* Misrouting’’[Title/Abstract]) OR (‘’Axon* Sprouting’’[Title/Abstract]) OR (‘’Axon Arbors’’[Title/Abstract]) OR (‘’Axon* Dispersion’’[Title/Abstract]) OR (‘’Axon* Deviation’’[Title/Abstract]) OR (‘’Reinnervation Mismatch’’[Title/Abstract]) OR (‘’Targeted Reinnervation’’[Title/Abstract]) OR (‘’Selective Regeneration’’[Title/Abstract]) OR (‘’’Selective Reinnervation’’[Title/Abstract]) OR (‘’Preferential Reinnervation’’[Title/Abstract]) OR (‘’Preferential Regeneration’’[Title/Abstract]) OR (‘’Random Reinnervation’’[Title/Abstract]) OR (‘’Failed Target Reinnervation’’[Title/Abstract]) OR (‘’Specific Reinnervation’’[Title/Abstract]) OR (‘’Aberrant Reinnervation’’[Title/Abstract])
	Labeling	Retrograde axonal tracing Anterograde axonal tracing	(‘’Radioactive Tracers’’[MeSH terms]) OR (‘’Neuroanatomical Tract- Tracing Techniques’’[MeSH terms]) OR (‘’Neuronal Tract- Tracers’’[MeSH terms]) OR (‘’Staining and Labeling’’[MeSH terms]) OR (‘’Fluorescent Antibody Technique’’[MeSH terms]) OR (‘’Neural Pathway Tracing’’[Title/Abstract]) OR (‘’Axon* Tracing’’[Title/Abstract]) OR (‘’Axon* Targeting’’[Title/Abstract]) OR (‘’Axon* Labeling’’[Title/Abstract]) OR (‘’Neural Tract-Tracers’’[Title/Abstract]) OR (‘’Retrograde Tracing’’[Title/Abstract]) OR (‘’Anterograde Tracing’’[Title/Abstract]) OR (‘’Retrograde Labeling’’[Title/Abstract]) OR (‘’Anterograde Labeling’’[Title/Abstract]) OR (‘’Fluoro-Gold’’[Title/Abstract]) OR (‘’DiI’’[Title/Abstract]) OR (‘’DiO’’[Title/Abstract]) OR (‘’Cholera Toxin B Subunit’’[Title/Abstract]) OR (’’Immunohistochemistry’’[MeSH Terms]) OR (‘’Neurofilament Proteins’’[MeSH Terms]) OR (‘’GAP-43’’[Title/Abstract]) OR (‘’βIII-Tubulin’’[Title/Abstract]) OR (‘’Fluoro-Gold’’[Title/Abstract]) OR (‘’Fast Blue’’[Title/Abstract]) OR (’’Nerve count’’[Title/Abstract]) OR (’’Axon count’’[Title/Abstract])
	Functionality	Sciatic, Peroneal and Tibial function index	(’’Enhanced Recovery After Surgery’’[MeSH terms]) OR (‘’Functional Status’’[MeSH terms]) OR (‘’Functional Performance’’[MeSH terms]) OR (‘’Recovery of Function’’[MeSH terms]) OR (‘’Behavioral Tests’’[MeSH Terms]) OR (‘’Sciatic Functional Index’’[Title/Abstract]) OR (’’SFI’’[Title/Abstract]) OR (‘’Pinch Test’’[Title/Abstract]) OR (‘’Withdrawal Reflex’’[Title/Abstract])
	Visualization	Microscopy Fluoromicroscopy	(‘’Electron Microscopy’’[MeSH Terms]) OR (‘’3D Imaging’’[Title/Abstract]) OR (‘’CLARITY’’[Title/Abstract]) OR (‘’iDISCO’’[Title/Abstract]) OR (‘’Light-Sheet Microscopy’’[Title/Abstract]) OR (‘’Confocal Microscopy’’[MeSH Terms]) OR (‘’Magnetic Resonance Imaging’’[MeSH Terms]) OR (‘’Diffusion Tensor Imaging’’[Title/Abstract]) OR (‘’DTI’’[Title/Abstract]) OR (‘’Optical Imaging’’[Title/Abstract]) OR (‘’Two-Photon Microscopy’’[Title/Abstract]) OR (’’Optogenetics’’[MeSH Terms]) OR (‘’Light-Activated Stimulation’’[Title/Abstract]) OR (‘’Axon* Functional Mapping’’[Title/Abstract])
	Electrostudies	Electrostimulation Tetanic force	(‘’Electrophysiology’’[MeSH Terms]) OR (‘’Nerve Conduction Studies’’[MeSH Terms]) OR (‘’Nerve Conduction Velocity’’[Title/Abstract]) OR (‘’Compound Muscle Action Potential’’[Title/Abstract]) OR (’’Electrostimulation’’[Title/Abstract]) OR (’’Electroneurography’’[Title/Abstract]) OR (’’EMNG’’[Title/Abstract])

**Table A2:** Step by Step synthesis of final search code for PubMed.

#1	Animals AND (Nerve Injury AND Nerve Repair AND NGC) AND Outcome
#2	(Nerve Injury AND Nerve Repair AND NGC) AND Outcome
#3	(Nerve Injury AND (Nerve Repair OR NGC) AND Structural Design AND Outcome
#4	Nerve Injury AND Axonal Guidance AND Outcome
#5	Nerve Repair AND Axonal Guidance AND Assessment
#6	NGC AND Structural Design AND Outcome
#7	((excludepreprints[Filter]) AND (fft[Filter]) AND (english[Filter])) NOT (’’review’’[Publication Type] OR ’’meta-analysis’’[Publication Type]) NOT (’’Spinal cord’’[Title]) NOT (‘’Brain’’[Title]) NOT (‘’Stroke’’[Title]) AND (medline[Filter]) AND (1000/1/1:2024/12/31[pdat])
#8	Final Search Code = (#1 OR #2 OR #3 OR #4 OR #5 OR #6) AND #7 = 2617

**Table A3:** Tabular summary of risk of bias assessment results across domains.

Study ID	Sequence Generation	Baseline Characteristics	Allocation Concealment	Random Housing	Blinded Intervention	Random Outcome Assessment	Blinded Outcome Assessment	Incomplete Outcome Data	Selective Outcome Reporting	Other Sources of Bias
Bodine-Fowler et al., 1997 [77]	Unclear	Low risk	Unclear	Unclear	High risk	Unclear	Unclear	High risk	High risk	Unclear
Valero Cabre et al., 2001 [78]	Unclear	Low risk	Unclear	Unclear	High risk	Low risk	Low risk	Low risk	Low risk	Unclear
Valero Cabre et al., 2004 [79]	Low risk	Low risk	Low risk	Low risk	Unclear	Low risk	Low risk	Low risk	Unclear	Unclear
De Ruiter et al., 2008 [80]	High risk	High risk	High risk	High risk	High risk	Low risk	Low risk	Low risk	Unclear	Unclear
Hu et al., 2009 [81]	Unclear	Low risk	Unclear	Unclear	High risk	High risk	Low risk	Low risk	Low risk	High risk
Huang et al., 2010 [82]	Unclear	Low risk	Unclear	Unclear	High risk	Low risk	Low risk	Low risk	Unclear	Unclear
Yao et al., 2010 [55]	Unclear	Low risk	Unclear	Unclear	High risk	Low risk	Low risk	Low risk	Low risk	Unclear
Zhang et al., 2011 [83]	Unclear	Low risk	Unclear	Unclear	High risk	Low risk	Low risk	Low risk	Low risk	Unclear
Daly et al., 2012 [31]	Unclear	Low risk	High risk	High risk	High risk	Low risk	Low risk	Unclear	Low risk	Unclear
Jiang et al., 2012 [84]	High risk	Unclear	High risk	Unclear	High risk	Low risk	Low risk	High risk	Unclear	Unclear
Yu et al., 2015 [85]	Unclear	Low risk	Unclear	Unclear	High risk	Unclear	Low risk	Unclear	Unclear	Unclear
Bozkurt et al., 2016 [86]	High risk	Unclear	High risk	Unclear	High risk	Low risk	Low risk	High risk	Unclear	Unclear
Van Neerven et al., 2017 [87]	High risk	Low risk	High risk	Unclear	High risk	Low risk	Low risk	Low risk	Low risk	Unclear
Zhu et al., 2017 [88]	Unclear	Low risk	High risk	Unclear	High risk	Low risk	Low risk	Unclear	Unclear	Unclear
Xue et al., 2018 [90]	Unclear	Low risk	High risk	Unclear	High risk	Low risk	Low risk	Low risk	Unclear	High risk
Liu et al., 2018 [89]	Unclear	High risk	High risk	Unclear	High risk	Unclear	Low risk	Unclear	Low risk	Unclear
Zhu et al., 2020 [91]	Unclear	Low risk	High risk	Unclear	High risk	Unclear	Low risk	Low risk	Unclear	Unclear
Lu et al., 2021 [92]	Unclear	Low risk	Unclear	Unclear	High risk	Unclear	Low risk	Low risk	Unclear	Unclear
Yang et al., 2023 [93]	Low risk	Low risk	Unclear	Unclear	High risk	Low risk	Low risk	Low risk	Unclear	High risk
