# Supplementary material for: Structural Features of Nerve Guidance Conduits and Scaffolds in Preventing Axonal Misdirection: A Systematic Review of Retrograde Tracing Studies
Source: Bioengineering (Basel). 2026 Feb 13;13(2):220. doi: 10.3390/bioengineering13020220 (PMC12937697; doi:10.3390/bioengineering13020220)
Supplement: Supplementary file 1 [file bioengineering-13-00220-s001.zip › Supplementary File.pdf]

Table S1. Final PubMed Search Query

((“animals”[MeSH Terms] OR “models, animal”[MeSH Terms] OR “animal experimentation”[MeSH Terms] OR “rodent”[Title/Abstract] OR “rat”[Title/Abstract] OR “mouse”[Title/Abstract] OR “mice”[Title/Abstract] OR “hamster”[Title/Abstract] OR “monkey”[Title/Abstract] OR “guinea pig”[Title/Abstract] OR “pig”[Title/Abstract] OR “rabbit”[Title/Abstract] OR “cat”[Title/Abstract] OR “dog”[Title/Abstract] OR “sheep”[Title/Abstract]) AND (((“peripheral nerve injuries”[MeSH Terms] OR “axotomy”[MeSH Terms] OR “nerve crush”[MeSH Terms] OR “nerve regeneration”[MeSH Terms] OR “nerve injury model”[Title/Abstract] OR “nerve crush”[Title/Abstract] OR “nerve crush model”[Title/Abstract] OR “axonotmesis”[Title/Abstract] OR “neurotmesis”[Title/Abstract] OR “nerve transection”[Title/Abstract] OR “nerve transection model”[Title/Abstract] OR “nerve cut”[Title/Abstract] OR “nerve gap”[Title/Abstract] OR “nerve defect”[Title/Abstract])) OR ((“nerve transfer”[MeSH Terms] OR “suture techniques”[MeSH Terms] OR “sutureless surgical procedures”[MeSH Terms] OR “Autografts”[MeSH Terms] OR “Neurotization”[Title/Abstract] OR “Autografts”[Title/Abstract] OR “nerve reconstruction”[Title/Abstract] OR “end to end suture”[Title/Abstract] OR “epineurial suture”[Title/Abstract] OR “graft repair”[Title/Abstract] OR “nerve repair”[Title/Abstract] OR “direct nerve repair”[Title/Abstract] OR “direct nerve suture”[Title/Abstract] OR “Microsuture”[Title/Abstract] OR “nerve surgery”[Title/Abstract])) OR ((“tissue scaffolds”[MeSH Terms] OR “nerve guidance conduits”[Title/Abstract] OR “nerve guidance channels”[Title/Abstract] OR “NGCs”[Title/Abstract] OR “nerve conduits”[Title/Abstract] OR “neural conduits”[Title/Abstract] OR “neural tubes”[Title/Abstract] OR “nerve tubes”[Title/Abstract] OR “nerve scaffold”[Title/Abstract] OR “neural scaffold”[Title/Abstract] OR “artificial nerve graft”[Title/Abstract] OR “artificial nerve conduit”[Title/Abstract] OR ((“fabric s”[All Fields] OR “fabricable”[All Fields] OR “fabricate”[All Fields] OR “fabricated”[All Fields] OR “fabricates”[All Fields] OR “fabricating”[All Fields] OR “fabrication”[All Fields] OR “fabrications”[All Fields] OR “textiles”[MeSH Terms] OR “textiles”[All Fields] OR “fabric”[All Fields] OR “fabrics”[All Fields]) AND “nerve graft”[Title/Abstract]) OR ((“synthetic”[All Fields] OR “synthetically”[All Fields] OR “synthetics”[All Fields] OR “synthetize”[All Fields] OR “synthetized”[All Fields] OR “synthetizing”[All Fields]) AND “nerve graft”[Title/Abstract]) OR “nerve guide”[Title/Abstract] OR “nerve connector”[Title/Abstract]))) AND ((“neurogenesis”[MeSH Terms] OR “neuronal outgrowth”[MeSH Terms] OR “axon guidance”[MeSH Terms] OR “axon fasciculation”[MeSH Terms] OR “axon guidance”[MeSH Terms] OR “axon* bundling”[Title/Abstract] OR “neurite fasciculation”[Title/Abstract] OR “axon guidance”[MeSH Terms] OR “neural guidance”[Title/Abstract] OR “axon* pruning”[Title/Abstract] OR “neurite pruning”[Title/Abstract] OR “neurite arborization”[Title/Abstract] OR “axon* arborization”[Title/Abstract] OR “synaptic pruning”[Title/Abstract] OR “axon* pathway”[Title/Abstract] OR “axon* misdirection”[Title/Abstract] OR “axon* direction”[Title/Abstract] OR (“axon*”[All Fields] AND “~~aligment~~alignment”[Title/Abstract]) OR (“axon*”[All Fields] AND “~~misaligment~~misalignment”[Title/Abstract]) OR “axon* orientation”[Title/Abstract] OR “axon* navigation”[Title/Abstract] OR “axon* misrouting”[Title/Abstract] OR “axon* sprouting”[Title/Abstract] OR “axon arbors”[Title/Abstract] OR “axon* dispersion”[Title/Abstract] OR (“axon*”[All Fields] AND “deviation”[Title/Abstract]) OR ((“reinnervate”[All Fields] OR “reinnervated”[All Fields] OR “reinnervates”[All Fields] OR “reinnervating”[All Fields] OR “reinnervation”[All Fields] OR “reinnervations”[All Fields]) AND “mismatch”[Title/Abstract]) OR “targeted reinnervation”[Title/Abstract] OR “selective regeneration”[Title/Abstract] OR “selective reinnervation”[Title/Abstract] OR “preferential reinnervation”[Title/Abstract] OR “preferential regeneration”[Title/Abstract] OR “random reinnervation”[Title/Abstract] OR ((“failed”[All Fields] OR “failing”[All Fields] OR “failings”[All Fields] OR “fails”[All Fields]) AND “target reinnervation”[Title/Abstract]) OR “specific reinnervation”[Title/Abstract] OR (“~~abberant~~aberrant”[All Fields] AND “reinnervation”[Title/Abstract])))) OR ((“animals”[MeSH Terms] OR “models, animal”[MeSH

Terms] OR “animal experimentation”[MeSH Terms] OR “rodent”[Title/Abstract] OR “rat”[Title/Abstract] OR “mouse”[Title/Abstract] OR “mice”[Title/Abstract] OR “hamster”[Title/Abstract] OR “monkey”[Title/Abstract] OR “guinea pig”[Title/Abstract] OR “pig”[Title/Abstract] OR “rabbit”[Title/Abstract] OR “cat”[Title/Abstract] OR “dog”[Title/Abstract] OR “sheep”[Title/Abstract]) AND (((“peripheral nerve injuries”[MeSH Terms] OR “axotomy”[MeSH Terms] OR “nerve crush”[MeSH Terms] OR “nerve regeneration”[MeSH Terms] OR “nerve injury model”[Title/Abstract] OR “nerve crush”[Title/Abstract] OR “nerve crush model”[Title/Abstract] OR “axonotmesis”[Title/Abstract] OR “neurotmesis”[Title/Abstract] OR “nerve transection”[Title/Abstract] OR “nerve transection model”[Title/Abstract] OR “nerve cut”[Title/Abstract] OR “nerve gap”[Title/Abstract] OR “nerve defect”[Title/Abstract])) OR ((“nerve transfer”[MeSH Terms] OR “suture techniques”[MeSH Terms] OR “sutureless surgical procedures”[MeSH Terms] OR “Autografts”[MeSH Terms] OR “Neurotization”[Title/Abstract] OR “Autografts”[Title/Abstract] OR “nerve reconstruction”[Title/Abstract] OR “end to end suture”[Title/Abstract] OR “epineurial suture”[Title/Abstract] OR “graft repair”[Title/Abstract] OR “nerve repair”[Title/Abstract] OR “direct nerve repair”[Title/Abstract] OR “direct nerve suture”[Title/Abstract] OR “Microsuture”[Title/Abstract] OR “nerve surgery”[Title/Abstract])) OR ((“tissue scaffolds”[MeSH Terms] OR “nerve guidance conduits”[Title/Abstract] OR “nerve guidance channels”[Title/Abstract] OR “NGCs”[Title/Abstract] OR “nerve conduits”[Title/Abstract] OR “neural conduits”[Title/Abstract] OR “neural tubes”[Title/Abstract] OR “nerve tubes”[Title/Abstract] OR “nerve scaffold”[Title/Abstract] OR “neural scaffold”[Title/Abstract] OR “artificial nerve graft”[Title/Abstract] OR “artificial nerve conduit”[Title/Abstract] OR ((“fabric s”[All Fields] OR “fabricable”[All Fields] OR “fabricate”[All Fields] OR “fabricated”[All Fields] OR “fabricates”[All Fields] OR “fabricating”[All Fields] OR “fabrication”[All Fields] OR “fabrications”[All Fields] OR “textiles”[MeSH Terms] OR “textiles”[All Fields] OR “fabric”[All Fields] OR “fabrics”[All Fields]) AND “nerve graft”[Title/Abstract]) OR ((“synthetic”[All Fields] OR “synthetically”[All Fields] OR “synthetics”[All Fields] OR “synthetize”[All Fields] OR “synthetized”[All Fields] OR “synthetizing”[All Fields]) AND “nerve graft”[Title/Abstract]) OR “nerve guide”[Title/Abstract] OR “nerve connector”[Title/Abstract]))) AND ((((“biomimetics”[MeSH Terms] OR “nanostructures”[MeSH Terms] OR ((“tissue guided”[All Fields] AND (“tissue s”[All Fields] OR “tissues”[MeSH Terms] OR “tissues”[All Fields] OR “tissue”[All Fields])) AND “regeneration”[MeSH Terms]) OR “Structure”[Title/Abstract] OR “Design”[Title/Abstract] OR “Property”[Title/Abstract] OR “structural design strategy”[Title/Abstract] OR “Architecture”[Title/Abstract] OR “Microarchitecture”[Title/Abstract] OR “Microstructure”[Title/Abstract] OR “Superstructure”[Title/Abstract] OR “Topography”[Title/Abstract] OR ((“lumen”[All Fields] OR “lumen s”[All Fields] OR “lumenal”[All Fields] OR “lumenally”[All Fields] OR “lumenization”[All Fields] OR “lumenized”[All Fields] OR “lumens”[All Fields]) AND “Topography”[Title/Abstract]) OR ((“lumen”[All Fields] OR “lumen s”[All Fields] OR “lumenal”[All Fields] OR “lumenally”[All Fields] OR “lumenization”[All Fields] OR “lumenized”[All Fields] OR “lumens”[All Fields]) AND “feature”[Title/Abstract]) OR ((“lumen”[All Fields] OR “lumen s”[All Fields] OR “lumenal”[All Fields] OR “lumenally”[All Fields] OR “lumenization”[All Fields] OR “lumenized”[All Fields] OR “lumens”[All Fields]) AND “surface feature”[Title/Abstract]) OR “structural feature”[Title/Abstract] OR “mechanical cues”[Title/Abstract] OR “topographic cues”[Title/Abstract] OR ((“topograph”[All Fields] OR “topographer”[All Fields] OR “topographers”[All Fields] OR “topographic”[All Fields] OR “topographical”[All Fields] OR “topographically”[All Fields] OR “topographics”[All Fields] OR “topographs”[All Fields]) AND “alignment”[Title/Abstract]) OR “Porous”[Title/Abstract] OR “Nanopores”[Title/Abstract]) AND “Grooved”[Title/Abstract]) OR “Microgrooved”[Title/Abstract] OR “Filled”[Title/Abstract] OR “luminal fillers”[Title/Abstract] OR “Nanofiber”[Title/Abstract] OR “filament fiber”[Title/Abstract] OR “Hydrogel”[Title/Abstract] OR “Nanosponge”[Title/Abstract] OR “Micropore”[Title/Abstract] OR “Multichannel”[Title/Abstract] OR “Microchannel”[Title/Abstract] OR “Multilumen”[Title/Abstract] OR “single channel”[Title/Abstract] OR “single lumen”[Title/Abstract] OR “single hollow”[Title/Abstract] OR “Multibranched”[Title/Abstract] OR

"Branched"[Title/Abstract] OR "Micropatterned"[Title/Abstract] OR "Bibranched"[Title/Abstract] OR "Tubular"[Title/Abstract] OR "Spiral"[Title/Abstract] OR "channel orientation"[Title/Abstract] OR "channel number"[Title/Abstract] OR "pores"[Title/Abstract] OR "holes"[Title/Abstract])) AND (((("radioactive tracers"[MeSH Terms] OR "neuroanatomical tract tracing techniques"[MeSH Terms] OR "neuronal tract tracers"[MeSH Terms] OR "staining and labeling"[MeSH Terms] OR "fluorescent antibody technique"[MeSH Terms] OR "neural pathway tracing"[Title/Abstract] OR "axon* tracing"[Title/Abstract] OR "axon* targeting"[Title/Abstract] OR "axon* labeling"[Title/Abstract] OR "neural tract tracers"[Title/Abstract] OR "retrograde tracing"[Title/Abstract] OR "anterograde tracing"[Title/Abstract] OR "retrograde labeling"[Title/Abstract] OR "anterograde labeling"[Title/Abstract] OR "fluoro gold"[Title/Abstract] OR "dii"[Title/Abstract] OR "dio"[Title/Abstract] OR "cholera toxin b subunit"[Title/Abstract] OR "immunohistochemistry"[MeSH Terms] OR "neurofilament proteins"[MeSH Terms] OR "gap 43"[Title/Abstract] OR "betaiii tubulin"[Title/Abstract] OR "fluoro gold"[Title/Abstract] OR "fast blue"[Title/Abstract] OR "nerve count"[Title/Abstract] OR "axon count"[Title/Abstract])) OR (("enhanced recovery after surgery"[MeSH Terms] OR "functional status"[MeSH Terms] OR "physical functional performance"[MeSH Terms] OR "recovery of function"[MeSH Terms] OR "behavior rating scale"[MeSH Terms] OR "sciatic functional index"[Title/Abstract] OR "sfi"[Title/Abstract] OR "pinch test"[Title/Abstract] OR "withdrawal reflex"[Title/Abstract])) OR (("microscopy, electron"[MeSH Terms] OR "3d imaging"[Title/Abstract] OR "clarity"[Title/Abstract] OR "idisco"[Title/Abstract] OR "light sheet microscopy"[Title/Abstract] OR "microscopy, confocal"[MeSH Terms] OR "magnetic resonance imaging"[MeSH Terms] OR "diffusion tensor imaging"[Title/Abstract] OR "dti"[Title/Abstract] OR "optical imaging"[Title/Abstract] OR "two photon microscopy"[Title/Abstract] OR "optogenetics"[MeSH Terms] OR ("light activated"[All Fields] AND "stimulation"[Title/Abstract]) OR ("axon*"[All Fields] AND "functional mapping"[Title/Abstract]))) OR (("electrophysiology"[MeSH Terms] OR "nerve conduction studies"[MeSH Terms] OR "nerve conduction velocity"[Title/Abstract] OR "compound muscle action potential"[Title/Abstract] OR "electrostimulation"[Title/Abstract] OR "electroneurography"[Title/Abstract] OR "emng"[Title/Abstract]))))) OR (("animals"[MeSH Terms] OR "models, animal"[MeSH Terms] OR "animal experimentation"[MeSH Terms] OR "rodent"[Title/Abstract] OR "rat"[Title/Abstract] OR "mouse"[Title/Abstract] OR "mice"[Title/Abstract] OR "hamster"[Title/Abstract] OR "monkey"[Title/Abstract] OR "guinea pig"[Title/Abstract] OR "pig"[Title/Abstract] OR "rabbit"[Title/Abstract] OR "cat"[Title/Abstract] OR "dog"[Title/Abstract] OR "sheep"[Title/Abstract]) AND (("peripheral nerve injuries"[MeSH Terms] OR "axotomy"[MeSH Terms] OR "nerve crush"[MeSH Terms] OR "nerve regeneration"[MeSH Terms] OR "nerve injury model"[Title/Abstract] OR "nerve crush"[Title/Abstract] OR "nerve crush model"[Title/Abstract] OR "axonotmesis"[Title/Abstract] OR "neurotmesis"[Title/Abstract] OR "nerve transection"[Title/Abstract] OR "nerve transection model"[Title/Abstract] OR "nerve cut"[Title/Abstract] OR "nerve gap"[Title/Abstract] OR "nerve defect"[Title/Abstract]) AND (("nerve transfer"[MeSH Terms] OR "suture techniques"[MeSH Terms] OR "sutureless surgical procedures"[MeSH Terms] OR "Autografts"[MeSH Terms] OR "Neurotization"[Title/Abstract] OR "Autografts"[Title/Abstract] OR "nerve reconstruction"[Title/Abstract] OR "end to end suture"[Title/Abstract] OR "epineurial suture"[Title/Abstract] OR "graft repair"[Title/Abstract] OR "nerve repair"[Title/Abstract] OR "direct nerve repair"[Title/Abstract] OR "direct nerve suture"[Title/Abstract] OR "Microsuture"[Title/Abstract] OR "nerve surgery"[Title/Abstract])) AND (("tissue scaffolds"[MeSH Terms] OR "nerve guidance conduits"[Title/Abstract] OR "nerve guidance channels"[Title/Abstract] OR "NGCs"[Title/Abstract] OR "nerve conduits"[Title/Abstract] OR "neural conduits"[Title/Abstract] OR "neural tubes"[Title/Abstract] OR "nerve tubes"[Title/Abstract] OR "nerve scaffold"[Title/Abstract] OR "neural scaffold"[Title/Abstract] OR "artificial nerve graft"[Title/Abstract] OR "artificial nerve conduit"[Title/Abstract] OR (("fabric s"[All Fields] OR "fabricable"[All Fields] OR "fabricate"[All Fields] OR "fabricated"[All Fields] OR "fabricates"[All Fields] OR "fabricating"[All Fields] OR "fabrication"[All Fields] OR "fabrications"[All Fields] OR "textiles"[MeSH Terms] OR "textiles"[All Fields] OR "fabric"[All Fields] OR "fabrics"[All Fields]) AND "nerve

graft”[Title/Abstract]) OR ((“synthetic”[All Fields] OR “synthetically”[All Fields] OR “synthetics”[All Fields] OR “synthetize”[All Fields] OR “synthetized”[All Fields] OR “synthetizing”[All Fields]) AND “nerve graft”[Title/Abstract]) OR “nerve guide”[Title/Abstract] OR “nerve connector”[Title/Abstract]))) AND ((((“radioactive tracers”[MeSH Terms] OR “neuroanatomical tract tracing techniques”[MeSH Terms] OR “neuronal tract tracers”[MeSH Terms] OR “staining and labeling”[MeSH Terms] OR “fluorescent antibody technique”[MeSH Terms] OR “neural pathway tracing”[Title/Abstract] OR “axon* tracing”[Title/Abstract] OR “axon* targeting”[Title/Abstract] OR “axon* labeling”[Title/Abstract] OR “neural tract tracers”[Title/Abstract] OR “retrograde tracing”[Title/Abstract] OR “anterograde tracing”[Title/Abstract] OR “retrograde labeling”[Title/Abstract] OR “anterograde labeling”[Title/Abstract] OR “fluoro gold”[Title/Abstract] OR “dii”[Title/Abstract] OR “dio”[Title/Abstract] OR “cholera toxin b subunit”[Title/Abstract] OR “immunohistochemistry”[MeSH Terms] OR “neurofilament proteins”[MeSH Terms] OR “gap 43”[Title/Abstract] OR “betaiii tubulin”[Title/Abstract] OR “fluoro gold”[Title/Abstract] OR “fast blue”[Title/Abstract] OR “nerve count”[Title/Abstract] OR “axon count”[Title/Abstract])) OR ((“enhanced recovery after surgery”[MeSH Terms] OR “functional status”[MeSH Terms] OR “physical functional performance”[MeSH Terms] OR “recovery of function”[MeSH Terms] OR “behavior rating scale”[MeSH Terms] OR “sciatic functional index”[Title/Abstract] OR “sfi”[Title/Abstract] OR “pinch test”[Title/Abstract] OR “withdrawal reflex”[Title/Abstract])) OR ((“microscopy, electron”[MeSH Terms] OR “3d imaging”[Title/Abstract] OR “clarity”[Title/Abstract] OR “idisco”[Title/Abstract] OR “light sheet microscopy”[Title/Abstract] OR “microscopy, confocal”[MeSH Terms] OR “magnetic resonance imaging”[MeSH Terms] OR “diffusion tensor imaging”[Title/Abstract] OR “dti”[Title/Abstract] OR “optical imaging”[Title/Abstract] OR “two photon microscopy”[Title/Abstract] OR “optogenetics”[MeSH Terms] OR (“light activated”[All Fields] AND “stimulation”[Title/Abstract]) OR (“axon*”[All Fields] AND “functional mapping”[Title/Abstract]))) OR ((“electrophysiology”[MeSH Terms] OR “nerve conduction studies”[MeSH Terms] OR “nerve conduction velocity”[Title/Abstract] OR “compound muscle action potential”[Title/Abstract] OR “electrostimulation”[Title/Abstract] OR “electroneurography”[Title/Abstract] OR “emng”[Title/Abstract]))))) OR ((“peripheral nerve injuries”[MeSH Terms] OR “axotomy”[MeSH Terms] OR “nerve crush”[MeSH Terms] OR “nerve regeneration”[MeSH Terms] OR “nerve injury model”[Title/Abstract] OR “nerve crush”[Title/Abstract] OR “nerve crush model”[Title/Abstract] OR “axonotmesis”[Title/Abstract] OR “neurotmesis”[Title/Abstract] OR “nerve transection”[Title/Abstract] OR “nerve transection model”[Title/Abstract] OR “nerve cut”[Title/Abstract] OR “nerve gap”[Title/Abstract] OR “nerve defect”[Title/Abstract]) AND ((“nerve transfer”[MeSH Terms] OR “suture techniques”[MeSH Terms] OR “sutureless surgical procedures”[MeSH Terms] OR “Autografts”[MeSH Terms] OR “Neurotization”[Title/Abstract] OR “Autografts”[Title/Abstract] OR “nerve reconstruction”[Title/Abstract] OR “end to end suture”[Title/Abstract] OR “epineurial suture”[Title/Abstract] OR “graft repair”[Title/Abstract] OR “nerve repair”[Title/Abstract] OR “direct nerve repair”[Title/Abstract] OR “direct nerve suture”[Title/Abstract] OR “Microsuture”[Title/Abstract] OR “nerve surgery”[Title/Abstract])) AND ((“tissue scaffolds”[MeSH Terms] OR “nerve guidance conduits”[Title/Abstract] OR “nerve guidance channels”[Title/Abstract] OR “NGCs”[Title/Abstract] OR “nerve conduits”[Title/Abstract] OR “neural conduits”[Title/Abstract] OR “neural tubes”[Title/Abstract] OR “nerve tubes”[Title/Abstract] OR “nerve scaffold”[Title/Abstract] OR “neural scaffold”[Title/Abstract] OR “artificial nerve graft”[Title/Abstract] OR “artificial nerve conduit”[Title/Abstract] OR ((“fabric s”[All Fields] OR “fabricable”[All Fields] OR “fabricate”[All Fields] OR “fabricated”[All Fields] OR “fabricates”[All Fields] OR “fabricating”[All Fields] OR “fabrication”[All Fields] OR “fabrications”[All Fields] OR “textiles”[MeSH Terms] OR “textiles”[All Fields] OR “fabric”[All Fields] OR “fabrics”[All Fields]) AND “nerve graft”[Title/Abstract]) OR ((“synthetic”[All Fields] OR “synthetically”[All Fields] OR “synthetics”[All Fields] OR “synthetize”[All Fields] OR “synthetized”[All Fields] OR “synthetizing”[All Fields]) AND “nerve graft”[Title/Abstract]) OR “nerve guide”[Title/Abstract] OR “nerve connector”[Title/Abstract])) AND ((((“radioactive tracers”[MeSH Terms] OR “neuroanatomical tract tracing techniques”[MeSH Terms] OR “neuronal

tract tracers"[MeSH Terms] OR "staining and labeling"[MeSH Terms] OR "fluorescent antibody technique"[MeSH Terms] OR "neural pathway tracing"[Title/Abstract] OR "axon* tracing"[Title/Abstract] OR "axon* targeting"[Title/Abstract] OR "axon* labeling"[Title/Abstract] OR "neural tract tracers"[Title/Abstract] OR "retrograde tracing"[Title/Abstract] OR "anterograde tracing"[Title/Abstract] OR "retrograde labeling"[Title/Abstract] OR "anterograde labeling"[Title/Abstract] OR "fluoro gold"[Title/Abstract] OR "dii"[Title/Abstract] OR "dio"[Title/Abstract] OR "cholera toxin b subunit"[Title/Abstract] OR "immunohistochemistry"[MeSH Terms] OR "neurofilament proteins"[MeSH Terms] OR "gap 43"[Title/Abstract] OR "betaiii tubulin"[Title/Abstract] OR "fluoro gold"[Title/Abstract] OR "fast blue"[Title/Abstract] OR "nerve count"[Title/Abstract] OR "axon count"[Title/Abstract])) OR (("enhanced recovery after surgery"[MeSH Terms] OR "functional status"[MeSH Terms] OR "physical functional performance"[MeSH Terms] OR "recovery of function"[MeSH Terms] OR "behavior rating scale"[MeSH Terms] OR "sciatic functional index"[Title/Abstract] OR "sfi"[Title/Abstract] OR "pinch test"[Title/Abstract] OR "withdrawal reflex"[Title/Abstract])) OR (("microscopy, electron"[MeSH Terms] OR "3d imaging"[Title/Abstract] OR "clarity"[Title/Abstract] OR "idisco"[Title/Abstract] OR "light sheet microscopy"[Title/Abstract] OR "microscopy, confocal"[MeSH Terms] OR "magnetic resonance imaging"[MeSH Terms] OR "diffusion tensor imaging"[Title/Abstract] OR "dti"[Title/Abstract] OR "optical imaging"[Title/Abstract] OR "two photon microscopy"[Title/Abstract] OR "optogenetics"[MeSH Terms] OR ("light activated"[All Fields] AND "stimulation"[Title/Abstract]) OR ("axon*"[All Fields] AND "functional mapping"[Title/Abstract]))) OR (("electrophysiology"[MeSH Terms] OR "nerve conduction studies"[MeSH Terms] OR "nerve conduction velocity"[Title/Abstract] OR "compound muscle action potential"[Title/Abstract] OR "electrostimulation"[Title/Abstract] OR "electroneurography"[Title/Abstract] OR "emng"[Title/Abstract]))))) OR (("peripheral nerve injuries"[MeSH Terms] OR "axotomy"[MeSH Terms] OR "nerve crush"[MeSH Terms] OR "nerve regeneration"[MeSH Terms] OR "nerve injury model"[Title/Abstract] OR "nerve crush"[Title/Abstract] OR "nerve crush model"[Title/Abstract] OR "axonotmesis"[Title/Abstract] OR "neurotmesis"[Title/Abstract] OR "nerve transection"[Title/Abstract] OR "nerve transection model"[Title/Abstract] OR "nerve cut"[Title/Abstract] OR "nerve gap"[Title/Abstract] OR "nerve defect"[Title/Abstract]) AND ((("nerve transfer"[MeSH Terms] OR "suture techniques"[MeSH Terms] OR "sutureless surgical procedures"[MeSH Terms] OR "Autografts"[MeSH Terms] OR "Neurotization"[Title/Abstract] OR "Autografts"[Title/Abstract] OR "nerve reconstruction"[Title/Abstract] OR "end to end suture"[Title/Abstract] OR "epineurial suture"[Title/Abstract] OR "graft repair"[Title/Abstract] OR "nerve repair"[Title/Abstract] OR "direct nerve repair"[Title/Abstract] OR "direct nerve suture"[Title/Abstract] OR "Microsuture"[Title/Abstract] OR "nerve surgery"[Title/Abstract])) OR (("tissue scaffolds"[MeSH Terms] OR "nerve guidance conduits"[Title/Abstract] OR "nerve guidance channels"[Title/Abstract] OR "NGCs"[Title/Abstract] OR "nerve conduits"[Title/Abstract] OR "neural conduits"[Title/Abstract] OR "neural tubes"[Title/Abstract] OR "nerve tubes"[Title/Abstract] OR "nerve scaffold"[Title/Abstract] OR "neural scaffold"[Title/Abstract] OR "artificial nerve graft"[Title/Abstract] OR "artificial nerve conduit"[Title/Abstract] OR (("fabric s"[All Fields] OR "fabricable"[All Fields] OR "fabricate"[All Fields] OR "fabricated"[All Fields] OR "fabricates"[All Fields] OR "fabricating"[All Fields] OR "fabrication"[All Fields] OR "fabrications"[All Fields] OR "textiles"[MeSH Terms] OR "textiles"[All Fields] OR "fabric"[All Fields] OR "fabrics"[All Fields]) AND "nerve graft"[Title/Abstract]) OR (("synthetic"[All Fields] OR "synthetically"[All Fields] OR "synthetics"[All Fields] OR "synthetize"[All Fields] OR "synthetized"[All Fields] OR "synthetizing"[All Fields]) AND "nerve graft"[Title/Abstract]) OR "nerve guide"[Title/Abstract] OR "nerve connector"[Title/Abstract]))) AND (((("biomimetics"[MeSH Terms] OR "nanostructures"[MeSH Terms] OR (("tissue guided"[All Fields] AND ("tissue s"[All Fields] OR "tissues"[MeSH Terms] OR "tissues"[All Fields] OR "tissue"[All Fields])) AND "regeneration"[MeSH Terms]) OR "Structure"[Title/Abstract] OR "Design"[Title/Abstract] OR "Property"[Title/Abstract] OR "structural design strategy"[Title/Abstract] OR "Architecture"[Title/Abstract] OR "Microarchitecture"[Title/Abstract] OR "Microstructure"[Title/Abstract] OR "Superstructure"[Title/Abstract] OR

"Topography"[Title/Abstract] OR (("lumen"[All Fields] OR "lumen s"[All Fields] OR "lumenal"[All Fields] OR "lumenally"[All Fields] OR "lumenization"[All Fields] OR "lumenized"[All Fields] OR "lumens"[All Fields]) AND "Topography"[Title/Abstract]) OR (("lumen"[All Fields] OR "lumen s"[All Fields] OR "lumenal"[All Fields] OR "lumenally"[All Fields] OR "lumenization"[All Fields] OR "lumenized"[All Fields] OR "lumens"[All Fields]) AND "feature"[Title/Abstract]) OR (("lumen"[All Fields] OR "lumen s"[All Fields] OR "lumenal"[All Fields] OR "lumenally"[All Fields] OR "lumenization"[All Fields] OR "lumenized"[All Fields] OR "lumens"[All Fields]) AND "surface feature"[Title/Abstract]) OR "structural feature"[Title/Abstract] OR "mechanical cues"[Title/Abstract] OR "topographic cues"[Title/Abstract] OR (("topograph"[All Fields] OR "topographer"[All Fields] OR "topographers"[All Fields] OR "topographic"[All Fields] OR "topographical"[All Fields] OR "topographically"[All Fields] OR "topographics"[All Fields] OR "topographs"[All Fields]) AND "alignment"[Title/Abstract]) OR "Porous"[Title/Abstract] OR "Nanopores"[Title/Abstract]) AND "Grooved"[Title/Abstract]) OR "Microgrooved"[Title/Abstract] OR "Filled"[Title/Abstract] OR "luminal fillers"[Title/Abstract] OR "Nanofiber"[Title/Abstract] OR "filament fiber"[Title/Abstract] OR "Hydrogel"[Title/Abstract] OR "Nanosponge"[Title/Abstract] OR "Micropore"[Title/Abstract] OR "Multichannel"[Title/Abstract] OR "Microchannel"[Title/Abstract] OR "Multilumen"[Title/Abstract] OR "single channel"[Title/Abstract] OR "single lumen"[Title/Abstract] OR "single hollow"[Title/Abstract] OR "Multibranched"[Title/Abstract] OR "Branched"[Title/Abstract] OR "Micropatterned"[Title/Abstract] OR "Bibranched"[Title/Abstract] OR "Tubular"[Title/Abstract] OR "Spiral"[Title/Abstract] OR "channel orientation"[Title/Abstract] OR "channel number"[Title/Abstract] OR "pores"[Title/Abstract] OR "holes"[Title/Abstract])) AND (((("radioactive tracers"[MeSH Terms] OR "neuroanatomical tract tracing techniques"[MeSH Terms] OR "neuronal tract tracers"[MeSH Terms] OR "staining and labeling"[MeSH Terms] OR "fluorescent antibody technique"[MeSH Terms] OR "neural pathway tracing"[Title/Abstract] OR "axon* tracing"[Title/Abstract] OR "axon* targeting"[Title/Abstract] OR "axon* labeling"[Title/Abstract] OR "neural tract tracers"[Title/Abstract] OR "retrograde tracing"[Title/Abstract] OR "anterograde tracing"[Title/Abstract] OR "retrograde labeling"[Title/Abstract] OR "anterograde labeling"[Title/Abstract] OR "fluoro gold"[Title/Abstract] OR "dii"[Title/Abstract] OR "dio"[Title/Abstract] OR "cholera toxin b subunit"[Title/Abstract] OR "immunohistochemistry"[MeSH Terms] OR "neurofilament proteins"[MeSH Terms] OR "gap 43"[Title/Abstract] OR "betaiii tubulin"[Title/Abstract] OR "fluoro gold"[Title/Abstract] OR "fast blue"[Title/Abstract] OR "nerve count"[Title/Abstract] OR "axon count"[Title/Abstract])) OR (("enhanced recovery after surgery"[MeSH Terms] OR "functional status"[MeSH Terms] OR "physical functional performance"[MeSH Terms] OR "recovery of function"[MeSH Terms] OR "behavior rating scale"[MeSH Terms] OR "sciatic functional index"[Title/Abstract] OR "sfi"[Title/Abstract] OR "pinch test"[Title/Abstract] OR "withdrawal reflex"[Title/Abstract])) OR (("microscopy, electron"[MeSH Terms] OR "3d imaging"[Title/Abstract] OR "clarity"[Title/Abstract] OR "idisco"[Title/Abstract] OR "light sheet microscopy"[Title/Abstract] OR "microscopy, confocal"[MeSH Terms] OR "magnetic resonance imaging"[MeSH Terms] OR "diffusion tensor imaging"[Title/Abstract] OR "dti"[Title/Abstract] OR "optical imaging"[Title/Abstract] OR "two photon microscopy"[Title/Abstract] OR "optogenetics"[MeSH Terms] OR ("light activated"[All Fields] AND "stimulation"[Title/Abstract]) OR ("axon*"[All Fields] AND "functional mapping"[Title/Abstract]))) OR (("electrophysiology"[MeSH Terms] OR "nerve conduction studies"[MeSH Terms] OR "nerve conduction velocity"[Title/Abstract] OR "compound muscle action potential"[Title/Abstract] OR "electrostimulation"[Title/Abstract] OR "electroneurography"[Title/Abstract] OR "emng"[Title/Abstract]))))) OR (("neurogenesis"[MeSH Terms] OR "neuronal outgrowth"[MeSH Terms] OR "axon guidance"[MeSH Terms] OR "axon fasciculation"[MeSH Terms] OR "axon guidance"[MeSH Terms] OR "axon* bundling"[Title/Abstract] OR "neurite fasciculation"[Title/Abstract] OR "axon guidance"[MeSH Terms] OR "neural guidance"[Title/Abstract] OR "axon* pruning"[Title/Abstract] OR "neurite pruning"[Title/Abstract] OR "neurite arborization"[Title/Abstract] OR "axon* arborization"[Title/Abstract] OR "synaptic pruning"[Title/Abstract] OR "axon*

pathway”[Title/Abstract] OR “axon* misdirection”[Title/Abstract] OR “axon* direction”[Title/Abstract] OR (“axon*”[All Fields] AND “alignment”[Title/Abstract]) OR (“axon*”[All Fields] AND “~~misaligment~~misalignment”[Title/Abstract]) OR “axon* orientation”[Title/Abstract] OR “axon* navigation”[Title/Abstract] OR “axon* misrouting”[Title/Abstract] OR “axon* sprouting”[Title/Abstract] OR “axon arbors”[Title/Abstract] OR “axon* dispersion”[Title/Abstract] OR (“axon*”[All Fields] AND “deviation”[Title/Abstract]) OR ((“reinnervate”[All Fields] OR “reinnervated”[All Fields] OR “reinnervates”[All Fields] OR “reinnervating”[All Fields] OR “reinnervation”[All Fields] OR “reinnervations”[All Fields]) AND “mismatch”[Title/Abstract]) OR “targeted reinnervation”[Title/Abstract] OR “selective regeneration”[Title/Abstract] OR “selective reinnervation”[Title/Abstract] OR “preferential reinnervation”[Title/Abstract] OR “preferential regeneration”[Title/Abstract] OR “random reinnervation”[Title/Abstract] OR ((“failed”[All Fields] OR “failing”[All Fields] OR “failings”[All Fields] OR “fails”[All Fields]) AND “target reinnervation”[Title/Abstract]) OR “specific reinnervation”[Title/Abstract] OR (“~~abberant~~aberrant”[All Fields] AND “reinnervation”[Title/Abstract])) AND ((((“radioactive tracers”[MeSH Terms] OR “neuroanatomical tract tracing techniques”[MeSH Terms] OR “neuronal tract tracers”[MeSH Terms] OR “staining and labeling”[MeSH Terms] OR “fluorescent antibody technique”[MeSH Terms] OR “neural pathway tracing”[Title/Abstract] OR “axon* tracing”[Title/Abstract] OR “axon* targeting”[Title/Abstract] OR “axon* labeling”[Title/Abstract] OR “neural tract tracers”[Title/Abstract] OR “retrograde tracing”[Title/Abstract] OR “anterograde tracing”[Title/Abstract] OR “retrograde labeling”[Title/Abstract] OR “anterograde labeling”[Title/Abstract] OR “fluoro gold”[Title/Abstract] OR “dii”[Title/Abstract] OR “dio”[Title/Abstract] OR “cholera toxin b subunit”[Title/Abstract] OR “immunohistochemistry”[MeSH Terms] OR “neurofilament proteins”[MeSH Terms] OR “gap 43”[Title/Abstract] OR “betaiii tubulin”[Title/Abstract] OR “fluoro gold”[Title/Abstract] OR “fast blue”[Title/Abstract] OR “nerve count”[Title/Abstract] OR “axon count”[Title/Abstract])) OR ((“enhanced recovery after surgery”[MeSH Terms] OR “functional status”[MeSH Terms] OR “physical functional performance”[MeSH Terms] OR “recovery of function”[MeSH Terms] OR “behavior rating scale”[MeSH Terms] OR “sciatic functional index”[Title/Abstract] OR “sfi”[Title/Abstract] OR “pinch test”[Title/Abstract] OR “withdrawal reflex”[Title/Abstract])) OR ((“microscopy, electron”[MeSH Terms] OR “3d imaging”[Title/Abstract] OR “clarity”[Title/Abstract] OR “idisco”[Title/Abstract] OR “light sheet microscopy”[Title/Abstract] OR “microscopy, confocal”[MeSH Terms] OR “magnetic resonance imaging”[MeSH Terms] OR “diffusion tensor imaging”[Title/Abstract] OR “dti”[Title/Abstract] OR “optical imaging”[Title/Abstract] OR “two photon microscopy”[Title/Abstract] OR “optogenetics”[MeSH Terms] OR (“light activated”[All Fields] AND “stimulation”[Title/Abstract]) OR (“axon*”[All Fields] AND “functional mapping”[Title/Abstract]))) OR ((“electrophysiology”[MeSH Terms] OR “nerve conduction studies”[MeSH Terms] OR “nerve conduction velocity”[Title/Abstract] OR “compound muscle action potential”[Title/Abstract] OR “electrostimulation”[Title/Abstract] OR “electroneurography”[Title/Abstract] OR “emng”[Title/Abstract])))) AND (((“peripheral nerve injuries”[MeSH Terms] OR “axotomy”[MeSH Terms] OR “nerve crush”[MeSH Terms] OR “nerve regeneration”[MeSH Terms] OR “nerve injury model”[Title/Abstract] OR “nerve crush”[Title/Abstract] OR “nerve crush model”[Title/Abstract] OR “axonotmesis”[Title/Abstract] OR “neurotmesis”[Title/Abstract] OR “nerve transection”[Title/Abstract] OR “nerve transection model”[Title/Abstract] OR “nerve cut”[Title/Abstract] OR “nerve gap”[Title/Abstract] OR “nerve defect”[Title/Abstract])) OR ((“nerve transfer”[MeSH Terms] OR “suture techniques”[MeSH Terms] OR “sutureless surgical procedures”[MeSH Terms] OR “Autografts”[MeSH Terms] OR “Neurotization”[Title/Abstract] OR “Autografts”[Title/Abstract] OR “nerve reconstruction”[Title/Abstract] OR “end to end suture”[Title/Abstract] OR “epineurial suture”[Title/Abstract] OR “graft repair”[Title/Abstract] OR “nerve repair”[Title/Abstract] OR “direct nerve repair”[Title/Abstract] OR “direct nerve suture”[Title/Abstract] OR “Microsuture”[Title/Abstract] OR “nerve surgery”[Title/Abstract])))) OR ((“tissue scaffolds”[MeSH Terms] OR “nerve guidance conduits”[Title/Abstract] OR “nerve guidance channels”[Title/Abstract] OR “NGCs”[Title/Abstract] OR “nerve conduits”[Title/Abstract] OR

“neural conduits”[Title/Abstract] OR “neural tubes”[Title/Abstract] OR “nerve tubes”[Title/Abstract] OR “nerve scaffold”[Title/Abstract] OR “neural scaffold”[Title/Abstract] OR “artificial nerve graft”[Title/Abstract] OR “artificial nerve conduit”[Title/Abstract] OR ((“fabric s”[All Fields] OR “fabricable”[All Fields] OR “fabricate”[All Fields] OR “fabricated”[All Fields] OR “fabricates”[All Fields] OR “fabricating”[All Fields] OR “fabrication”[All Fields] OR “fabrications”[All Fields] OR “textiles”[MeSH Terms] OR “textiles”[All Fields] OR “fabric”[All Fields] OR “fabrics”[All Fields]) AND “nerve graft”[Title/Abstract]) OR ((“synthetic”[All Fields] OR “synthetically”[All Fields] OR “synthetics”[All Fields] OR “synthetize”[All Fields] OR “synthetized”[All Fields] OR “synthetizing”[All Fields]) AND “nerve graft”[Title/Abstract]) OR “nerve guide”[Title/Abstract] OR “nerve connector”[Title/Abstract]) AND (((“neurogenesis”[MeSH Terms] OR “neuronal outgrowth”[MeSH Terms] OR “axon guidance”[MeSH Terms] OR “axon fasciculation”[MeSH Terms] OR “axon guidance”[MeSH Terms] OR “axon* bundling”[Title/Abstract] OR “neurite fasciculation”[Title/Abstract] OR “axon guidance”[MeSH Terms] OR “neural guidance”[Title/Abstract] OR “axon* pruning”[Title/Abstract] OR “neurite pruning”[Title/Abstract] OR “neurite arborization”[Title/Abstract] OR “axon* arborization”[Title/Abstract] OR “synaptic pruning”[Title/Abstract] OR “axon* pathway”[Title/Abstract] OR “axon* misdirection”[Title/Abstract] OR “axon* direction”[Title/Abstract] OR (“axon*”[All Fields] AND “alignment”[Title/Abstract]) OR (“axon*”[All Fields] AND “~~misaligment~~misalignment”[Title/Abstract]) OR “axon* orientation”[Title/Abstract] OR “axon* navigation”[Title/Abstract] OR “axon* misrouting”[Title/Abstract] OR “axon* sprouting”[Title/Abstract] OR “axon arbors”[Title/Abstract] OR “axon* dispersion”[Title/Abstract] OR (“axon*”[All Fields] AND “deviation”[Title/Abstract]) OR ((“reinnervate”[All Fields] OR “reinnervated”[All Fields] OR “reinnervates”[All Fields] OR “reinnervating”[All Fields] OR “reinnervation”[All Fields] OR “reinnervations”[All Fields]) AND “mismatch”[Title/Abstract]) OR “targeted reinnervation”[Title/Abstract] OR “selective regeneration”[Title/Abstract] OR “selective reinnervation”[Title/Abstract] OR “preferential reinnervation”[Title/Abstract] OR “preferential regeneration”[Title/Abstract] OR “random reinnervation”[Title/Abstract] OR ((“failed”[All Fields] OR “failing”[All Fields] OR “failings”[All Fields] OR “fails”[All Fields]) AND “target reinnervation”[Title/Abstract]) OR “specific reinnervation”[Title/Abstract] OR (“~~abberant~~aberrant”[All Fields] AND “reinnervation”[Title/Abstract]))) OR ((((“biomimetics”[MeSH Terms] OR “nanostructures”[MeSH Terms] OR ((“tissue guided”[All Fields] AND (“tissue s”[All Fields] OR “tissues”[MeSH Terms] OR “tissues”[All Fields] OR “tissue”[All Fields])) AND “regeneration”[MeSH Terms]) OR “Structure”[Title/Abstract] OR “Design”[Title/Abstract] OR “Property”[Title/Abstract] OR “structural design strategy”[Title/Abstract] OR “Architecture”[Title/Abstract] OR “Microarchitecture”[Title/Abstract] OR “Microstructure”[Title/Abstract] OR “Superstructure”[Title/Abstract] OR “Topography”[Title/Abstract] OR ((“lumen”[All Fields] OR “lumen s”[All Fields] OR “lumenal”[All Fields] OR “lumenally”[All Fields] OR “lumenization”[All Fields] OR “lumenized”[All Fields] OR “lumens”[All Fields]) AND “Topography”[Title/Abstract]) OR ((“lumen”[All Fields] OR “lumen s”[All Fields] OR “lumenal”[All Fields] OR “lumenally”[All Fields] OR “lumenization”[All Fields] OR “lumenized”[All Fields] OR “lumens”[All Fields]) AND “feature”[Title/Abstract]) OR ((“lumen”[All Fields] OR “lumen s”[All Fields] OR “lumenal”[All Fields] OR “lumenally”[All Fields] OR “lumenization”[All Fields] OR “lumenized”[All Fields] OR “lumens”[All Fields]) AND “surface feature”[Title/Abstract]) OR “structural feature”[Title/Abstract] OR “mechanical cues”[Title/Abstract] OR “topographic cues”[Title/Abstract] OR ((“topograph”[All Fields] OR “topographer”[All Fields] OR “topographers”[All Fields] OR “topographic”[All Fields] OR “topographical”[All Fields] OR “topographically”[All Fields] OR “topographics”[All Fields] OR “topographs”[All Fields]) AND “alignment”[Title/Abstract]) OR “Porous”[Title/Abstract] OR “Nanopores”[Title/Abstract]) AND “Grooved”[Title/Abstract]) OR “Microgrooved”[Title/Abstract] OR “Filled”[Title/Abstract] OR “luminal fillers”[Title/Abstract] OR “Nanofiber”[Title/Abstract] OR “filament fiber”[Title/Abstract] OR “Hydrogel”[Title/Abstract] OR “Nanosponge”[Title/Abstract] OR “Micropore”[Title/Abstract] OR “Multichannel”[Title/Abstract] OR “Microchannel”[Title/Abstract] OR “Multilumen”[Title/Abstract] OR “single channel”[Title/Abstract] OR “single

lumen"[Title/Abstract] OR "single hollow"[Title/Abstract] OR "Multibranched"[Title/Abstract] OR "Branched"[Title/Abstract] OR "Micropatterned"[Title/Abstract] OR "Bibranched"[Title/Abstract] OR "Tubular"[Title/Abstract] OR "Spiral"[Title/Abstract] OR "channel orientation"[Title/Abstract] OR "channel number"[Title/Abstract] OR "pores"[Title/Abstract] OR "holes"[Title/Abstract]) AND (((("radioactive tracers"[MeSH Terms] OR "neuroanatomical tract tracing techniques"[MeSH Terms] OR "neuronal tract tracers"[MeSH Terms] OR "staining and labeling"[MeSH Terms] OR "fluorescent antibody technique"[MeSH Terms] OR "neural pathway tracing"[Title/Abstract] OR "axon* tracing"[Title/Abstract] OR "axon* targeting"[Title/Abstract] OR "axon* labeling"[Title/Abstract] OR "neural tract tracers"[Title/Abstract] OR "retrograde tracing"[Title/Abstract] OR "anterograde tracing"[Title/Abstract] OR "retrograde labeling"[Title/Abstract] OR "anterograde labeling"[Title/Abstract] OR "fluoro gold"[Title/Abstract] OR "dii"[Title/Abstract] OR "dio"[Title/Abstract] OR "cholera toxin b subunit"[Title/Abstract] OR "immunohistochemistry"[MeSH Terms] OR "neurofilament proteins"[MeSH Terms] OR "gap 43"[Title/Abstract] OR "betaiii tubulin"[Title/Abstract] OR "fluoro gold"[Title/Abstract] OR "fast blue"[Title/Abstract] OR "nerve count"[Title/Abstract] OR "axon count"[Title/Abstract])) OR (("enhanced recovery after surgery"[MeSH Terms] OR "functional status"[MeSH Terms] OR "physical functional performance"[MeSH Terms] OR "recovery of function"[MeSH Terms] OR "behavior rating scale"[MeSH Terms] OR "sciatic functional index"[Title/Abstract] OR "sfi"[Title/Abstract] OR "pinch test"[Title/Abstract] OR "withdrawal reflex"[Title/Abstract])) OR (("microscopy, electron"[MeSH Terms] OR "3d imaging"[Title/Abstract] OR "clarity"[Title/Abstract] OR "idisco"[Title/Abstract] OR "light sheet microscopy"[Title/Abstract] OR "microscopy, confocal"[MeSH Terms] OR "magnetic resonance imaging"[MeSH Terms] OR "diffusion tensor imaging"[Title/Abstract] OR "dti"[Title/Abstract] OR "optical imaging"[Title/Abstract] OR "two photon microscopy"[Title/Abstract] OR "optogenetics"[MeSH Terms] OR ("light activated"[All Fields] AND "stimulation"[Title/Abstract]) OR ("axon*"[All Fields] AND "functional mapping"[Title/Abstract]))) OR (("electrophysiology"[MeSH Terms] OR "nerve conduction studies"[MeSH Terms] OR "nerve conduction velocity"[Title/Abstract] OR "compound muscle action potential"[Title/Abstract] OR "electrostimulation"[Title/Abstract] OR "electroneurography"[Title/Abstract] OR "emng"[Title/Abstract]))))))) AND ((excludepreprints[Filter]) AND (fft[Filter]) AND (english[Filter])) NOT (''review''[Publication Type] OR ''meta-analysis''[Publication Type]) NOT (''Spinal cord''[Title]) NOT (''Brain''[Title]) NOT (''Stroke''[Title]) AND (medline[Filter]) AND (1000/1/1:2024/12/31[pdat])

Table S2. AI-~~-~~generated translation of PubMed ~~Pubmed~~ search code ~~quary~~query for EBSCO discovery service.

("peripheral nerve injuries" OR "axotomy" OR "nerve crush" OR "nerve regeneration" OR "nerve injury model" OR "nerve crush model" OR "axonotmesis" OR "neurotmesis" OR "nerve transection" OR "nerve gap" OR "nerve defect") AND ("nerve transfer" OR "suture techniques" OR "Autografts" OR "nerve reconstruction" OR "end-to-end suture" OR "graft repair" OR "direct nerve repair" OR "m~~M~~icrosuture" OR "nerve surgery") AND ("nerve guidance conduits" OR "NGCs" OR "nerve scaffold" OR "artificial nerve conduit" OR "neural scaffold" OR "tissue scaffolds") AND ("axon guidance" OR "neuronal outgrowth" OR "axon dispersion" OR "axon sprouting" OR "targeted reinnervation" OR "selective regeneration" OR "specific reinnervation") AND ("sciatic nerve" OR "peroneal nerve" OR "tibial nerve" OR "brachial plexus" OR "median nerve" OR "ulnar nerve" OR "radial nerve" OR "femoral nerve" OR "facial nerve") AND (animal models OR rat OR mouse OR hamster OR monkey OR guinea pig OR rabbit OR cat OR dog OR sheep) AND (published from: 1000 to 2024).
